# Supplementary figures and images for: Multi-targeted therapy resistance via drug-induced secretome fucosylation
Source: eLife. 2023 Mar 24;12:e75191. doi: 10.7554/eLife.75191 (PMC10089660; doi:10.7554/eLife.75191)

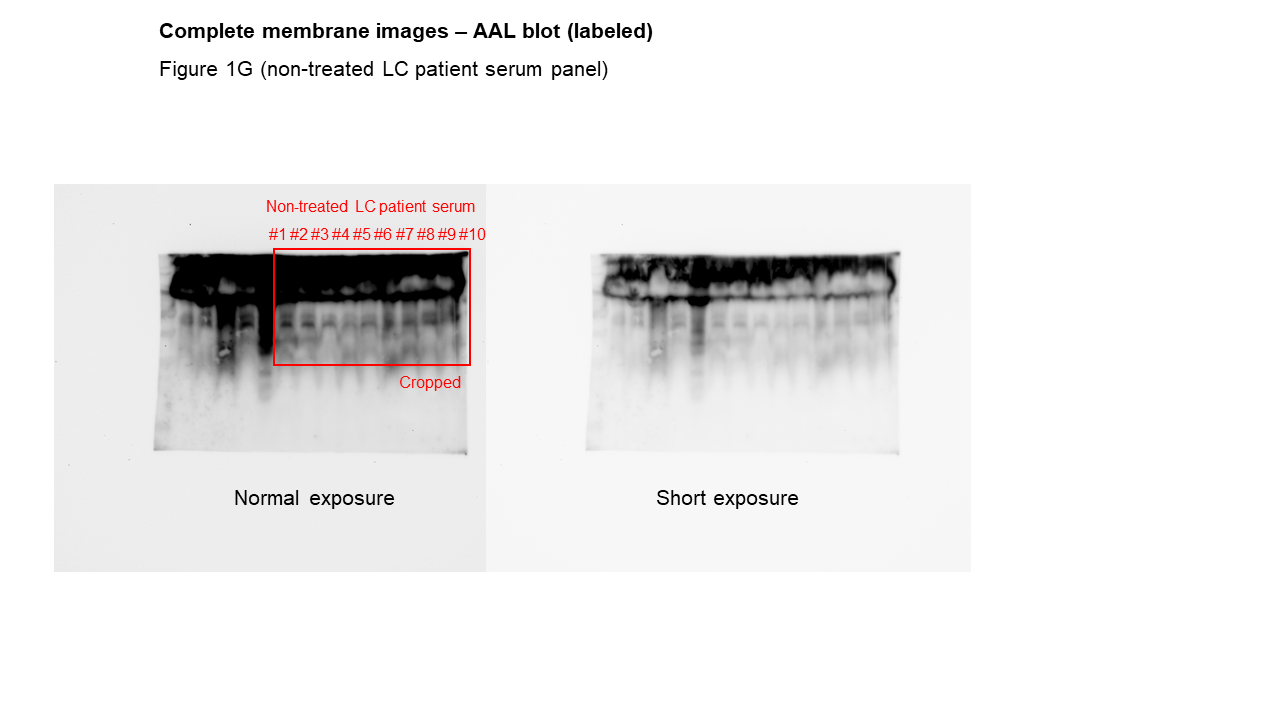

Supplement: Figure 1—source data 1. [file elife-75191-fig1-data1.zip › Figure 1/Figure 1G_1 (labeled).tif]

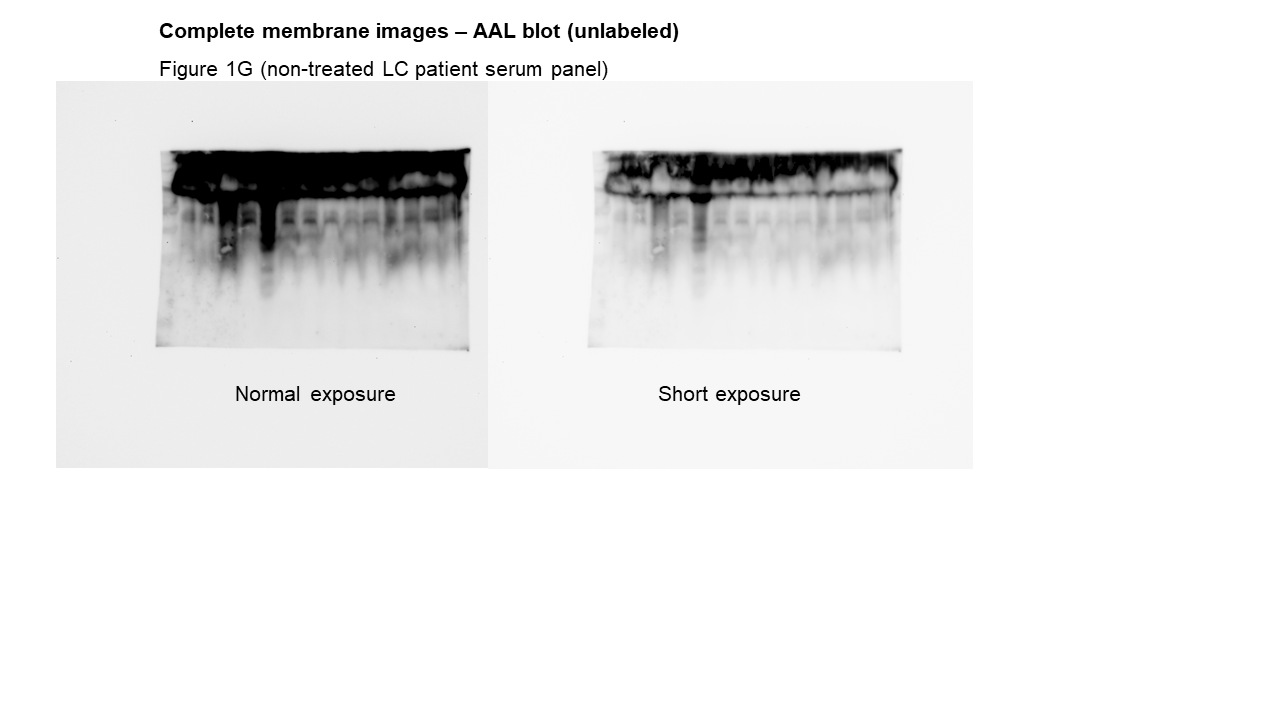

Supplement: Figure 1—source data 1. [file elife-75191-fig1-data1.zip › Figure 1/Figure 1G_1 (unlabeled).tif]

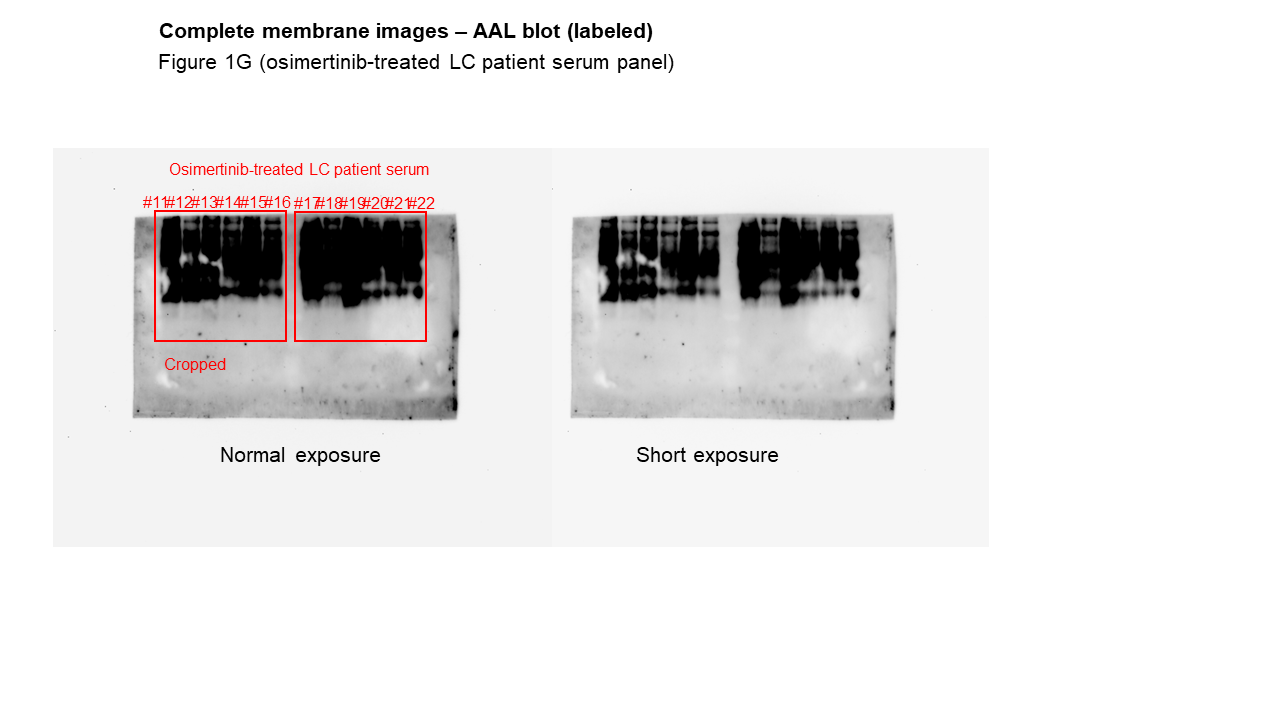

Supplement: Figure 1—source data 1. [file elife-75191-fig1-data1.zip › Figure 1/Figure 1G_2 (labeled).tif]

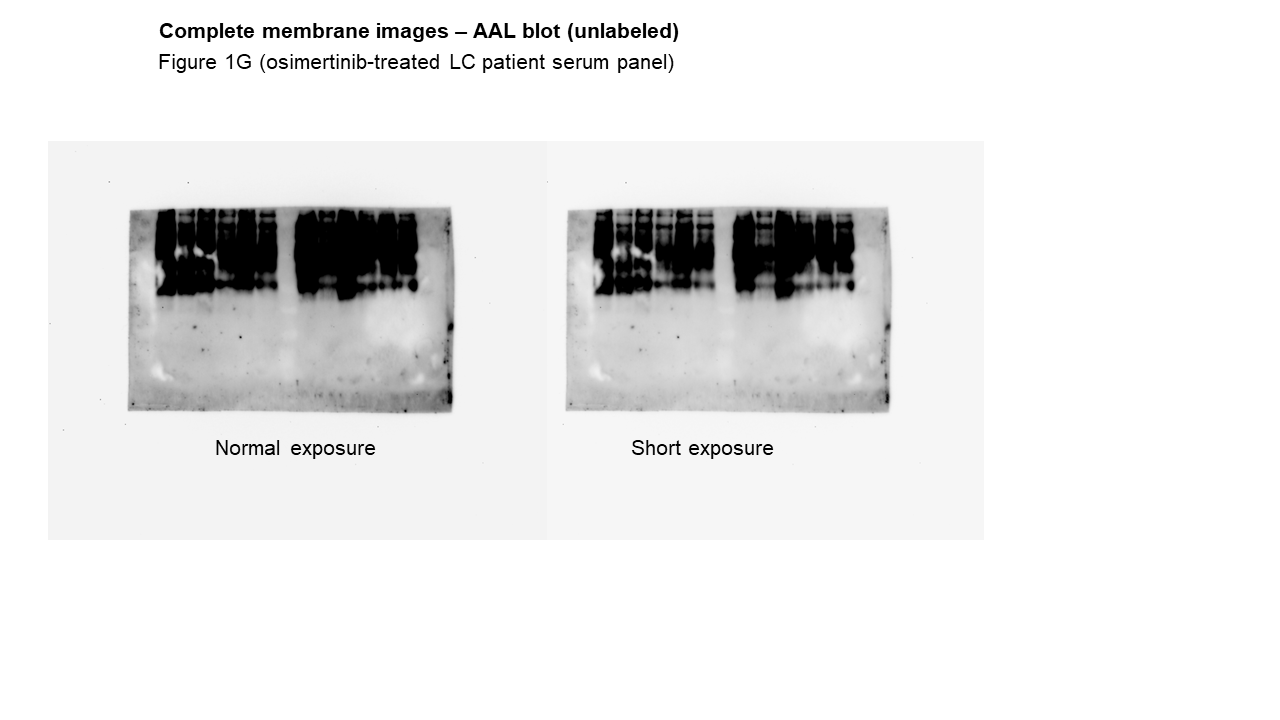

Supplement: Figure 1—source data 1. [file elife-75191-fig1-data1.zip › Figure 1/Figure 1G_2 (unlabeled).tif]

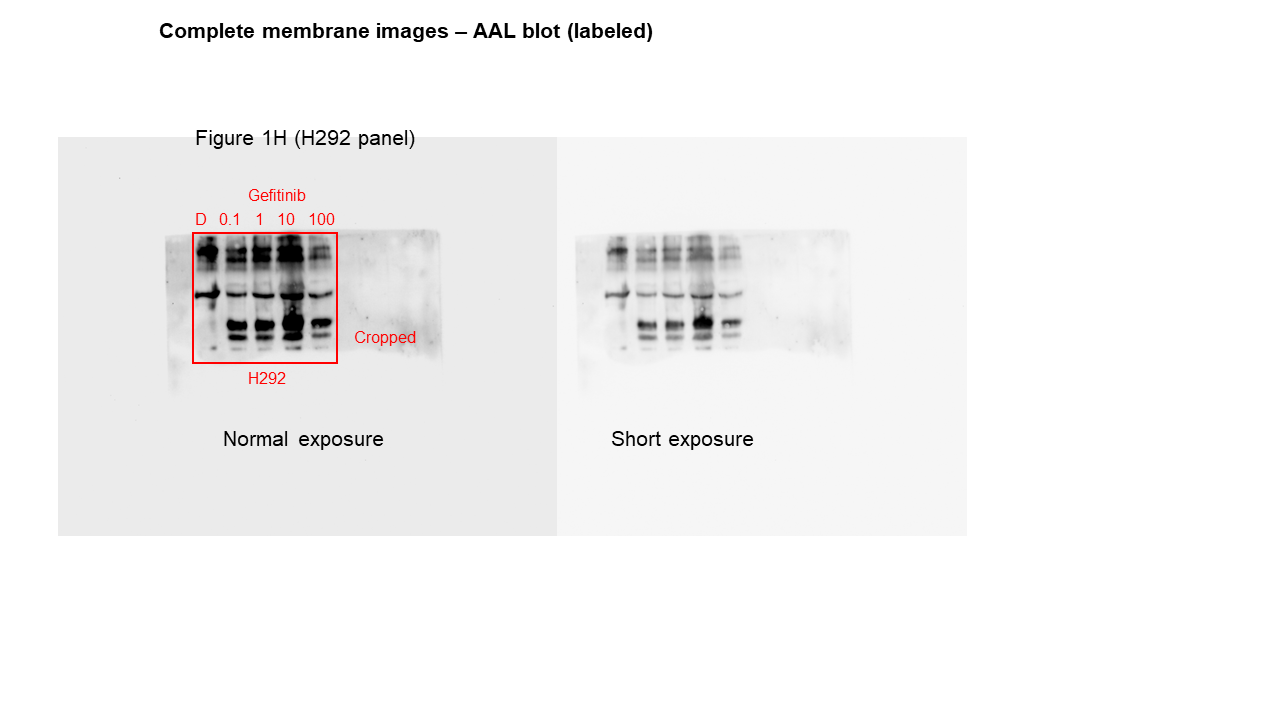

Supplement: Figure 1—source data 1. [file elife-75191-fig1-data1.zip › Figure 1/Figure 1H_1 (labeled).tif]

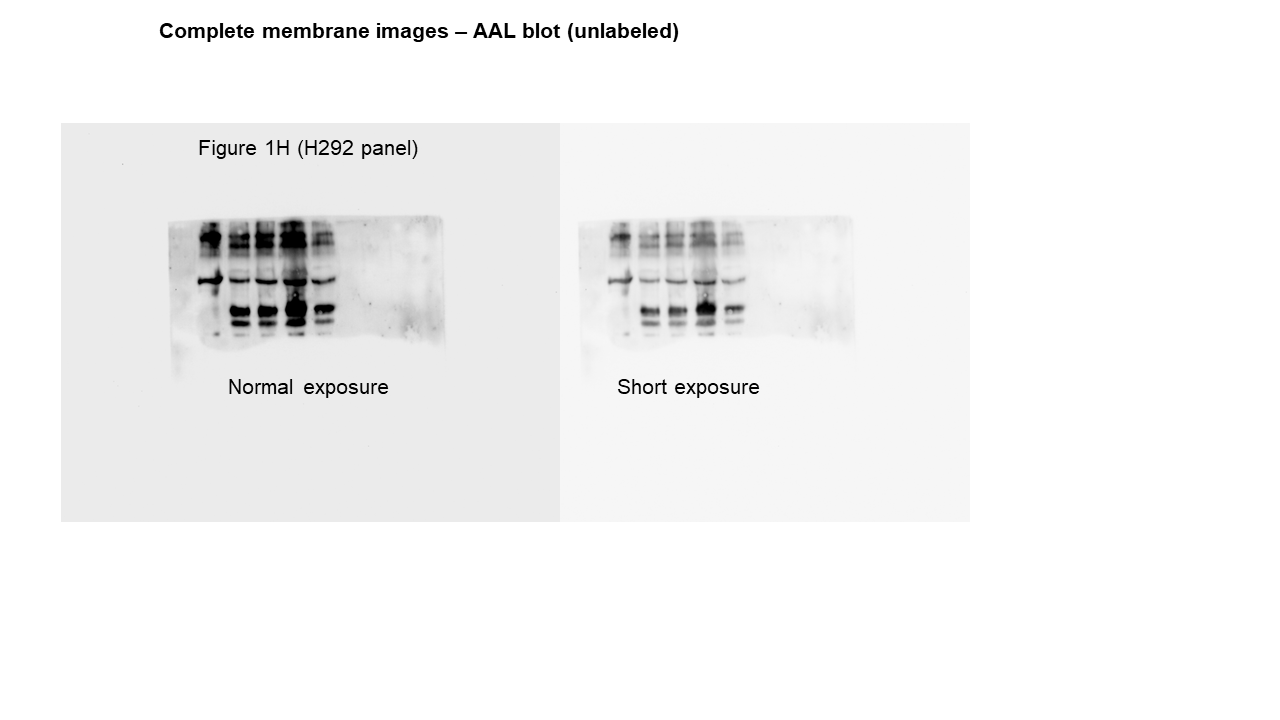

Supplement: Figure 1—source data 1. [file elife-75191-fig1-data1.zip › Figure 1/Figure 1H_1 (unlabeled).tif]

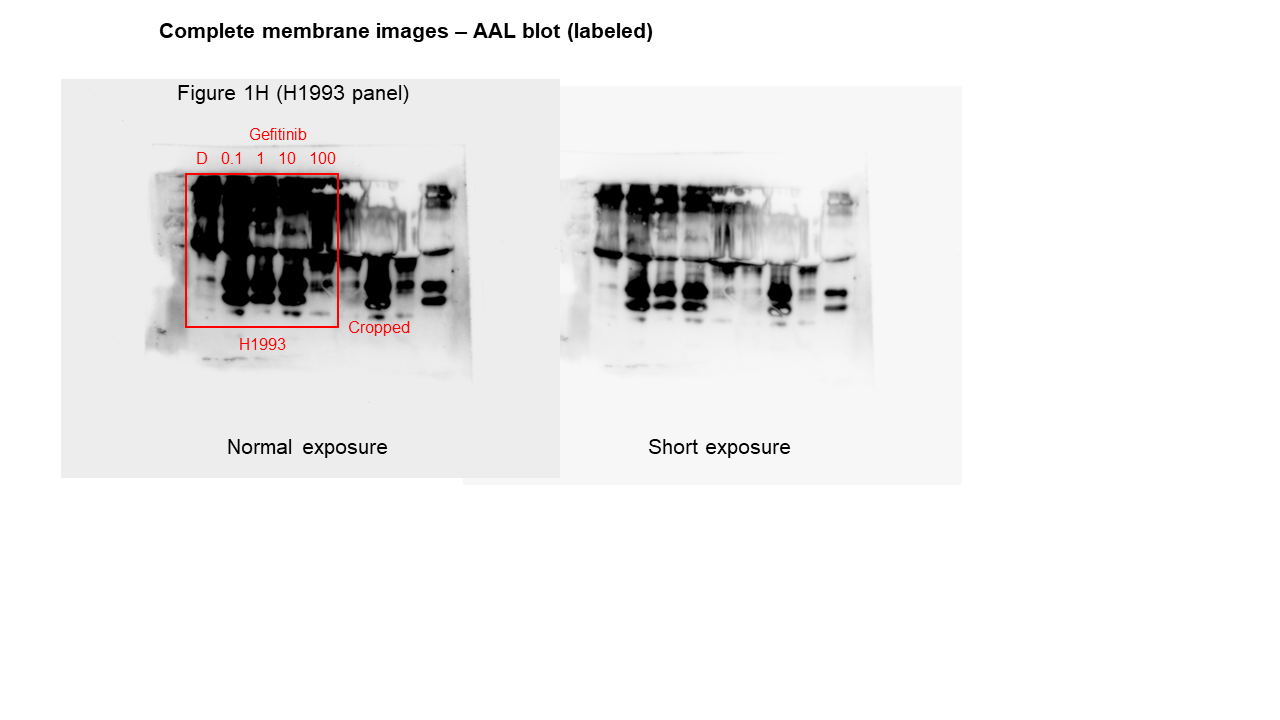

Supplement: Figure 1—source data 1. [file elife-75191-fig1-data1.zip › Figure 1/Figure 1H_2 (labeled).tif]

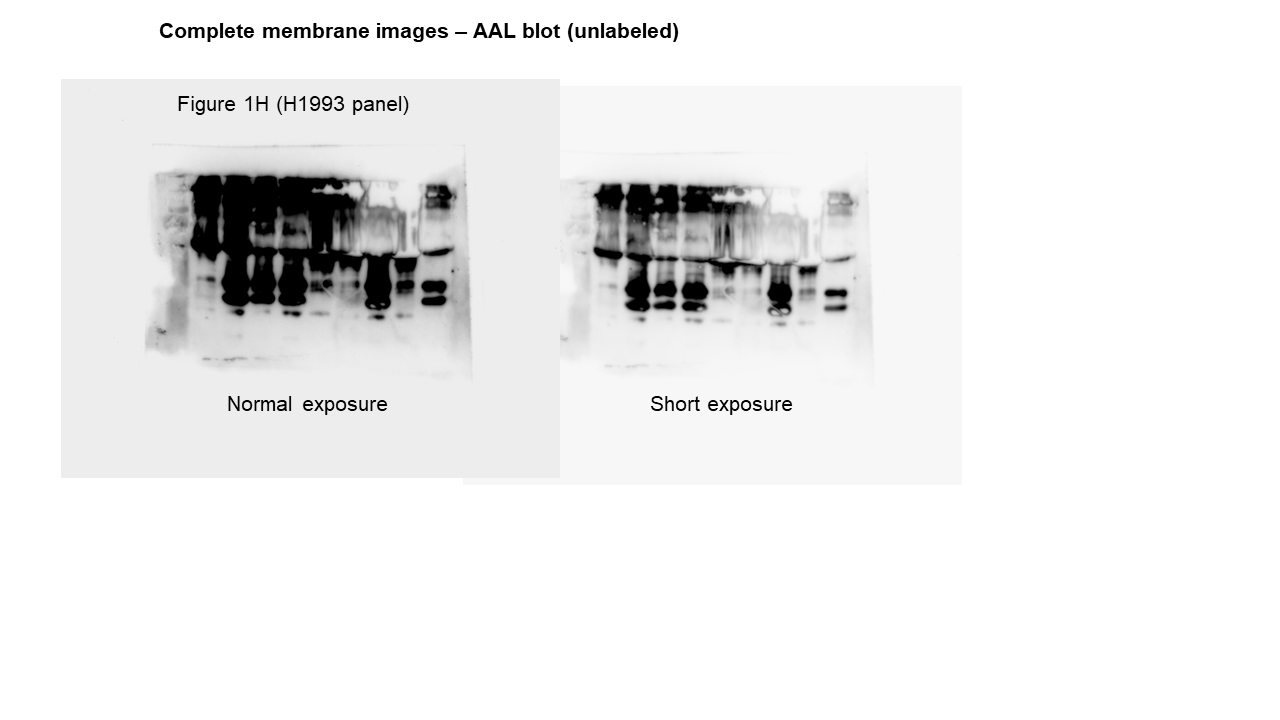

Supplement: Figure 1—source data 1. [file elife-75191-fig1-data1.zip › Figure 1/Figure 1H_2 (unlabeled).tif]

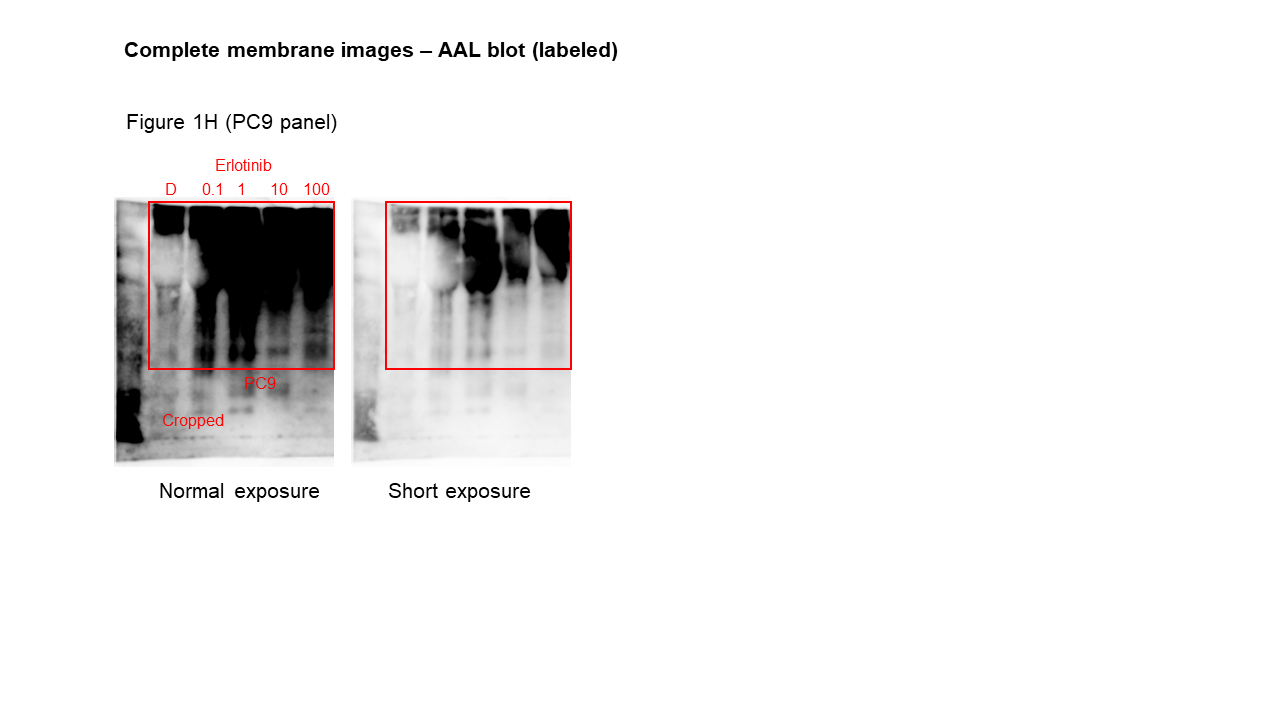

Supplement: Figure 1—source data 1. [file elife-75191-fig1-data1.zip › Figure 1/Figure 1H_3 (labeled).tif]

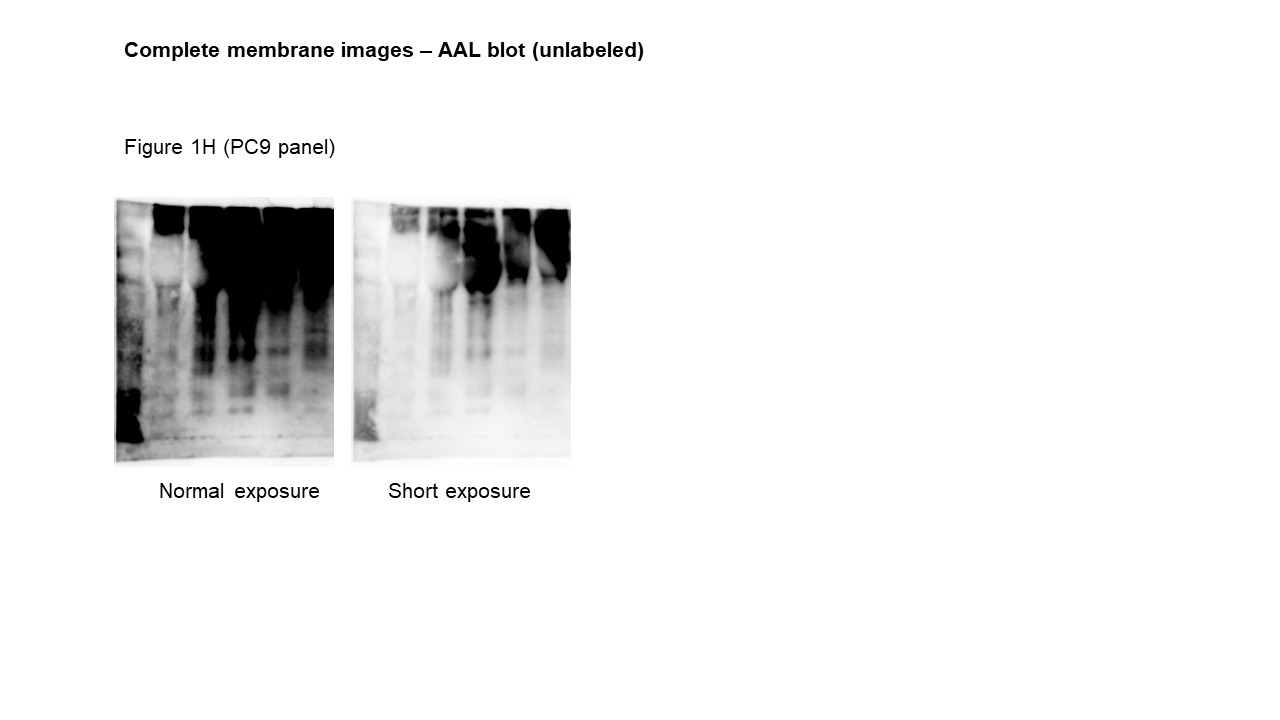

Supplement: Figure 1—source data 1. [file elife-75191-fig1-data1.zip › Figure 1/Figure 1H_3 (unlabeled).tif]

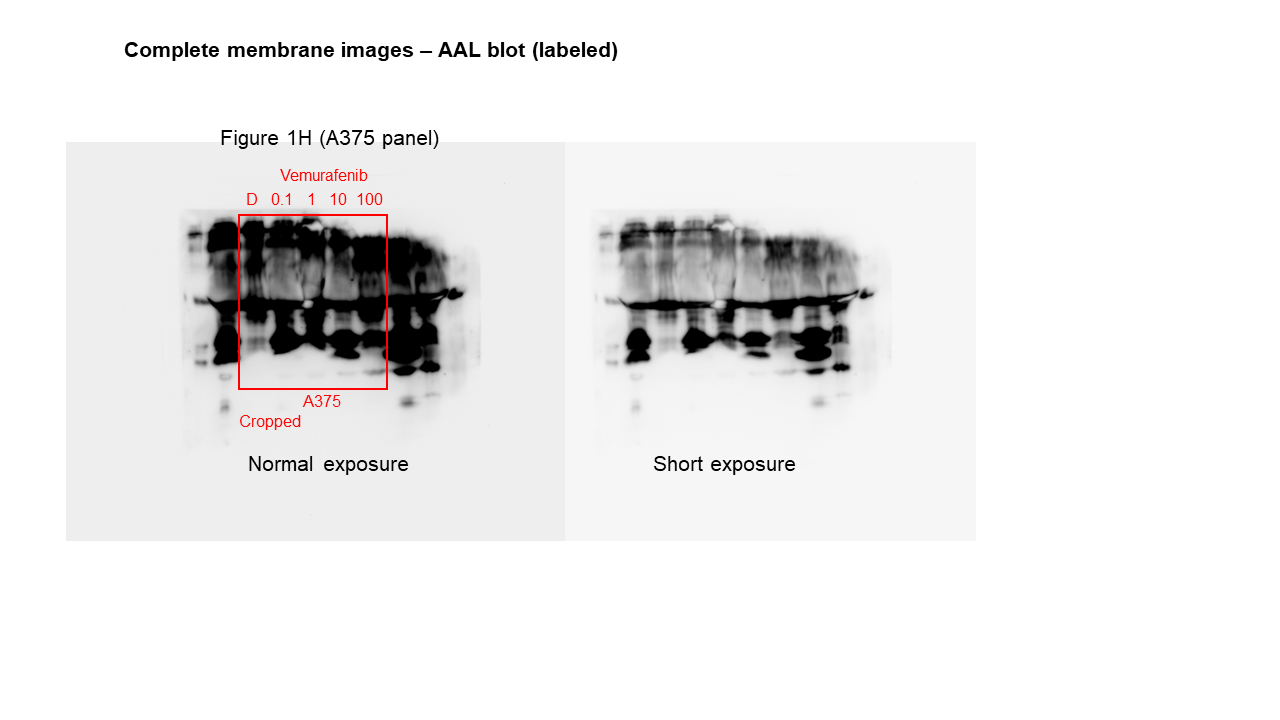

Supplement: Figure 1—source data 1. [file elife-75191-fig1-data1.zip › Figure 1/Figure 1H_4 (labeled).tif]

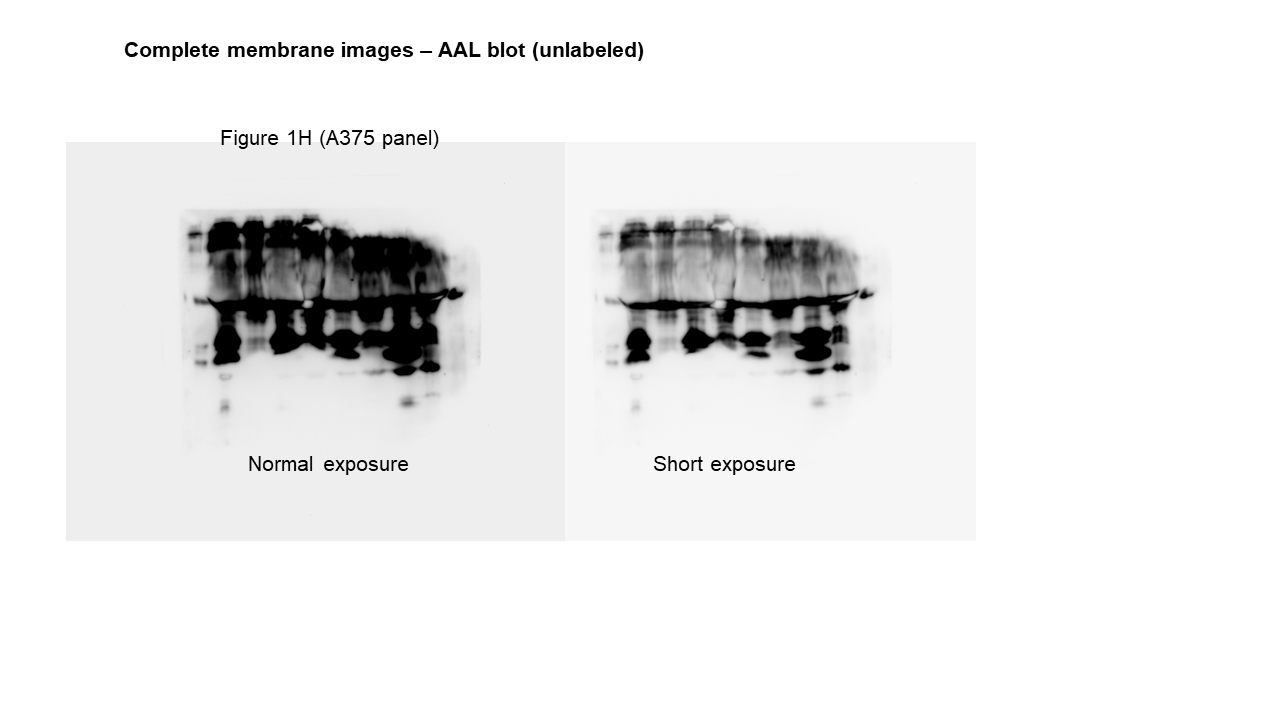

Supplement: Figure 1—source data 1. [file elife-75191-fig1-data1.zip › Figure 1/Figure 1H_4 (unlabeled).tif]

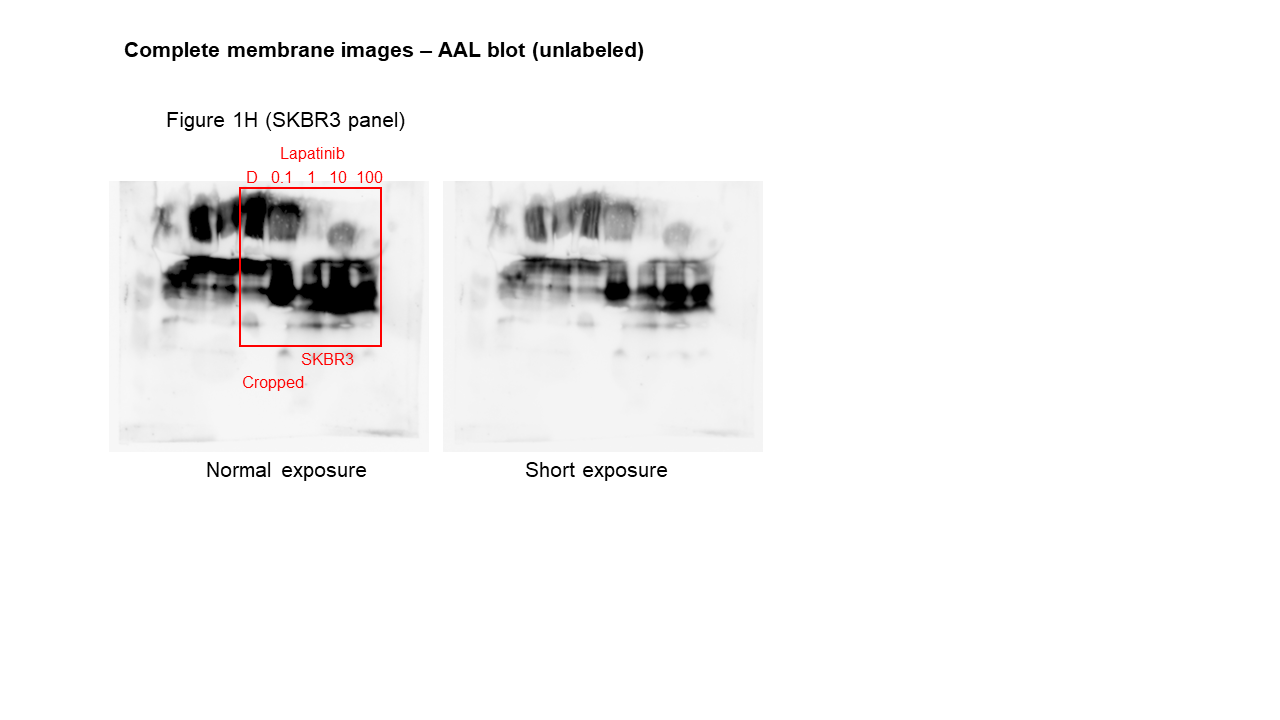

Supplement: Figure 1—source data 1. [file elife-75191-fig1-data1.zip › Figure 1/Figure 1H_5 (labeled).tif]

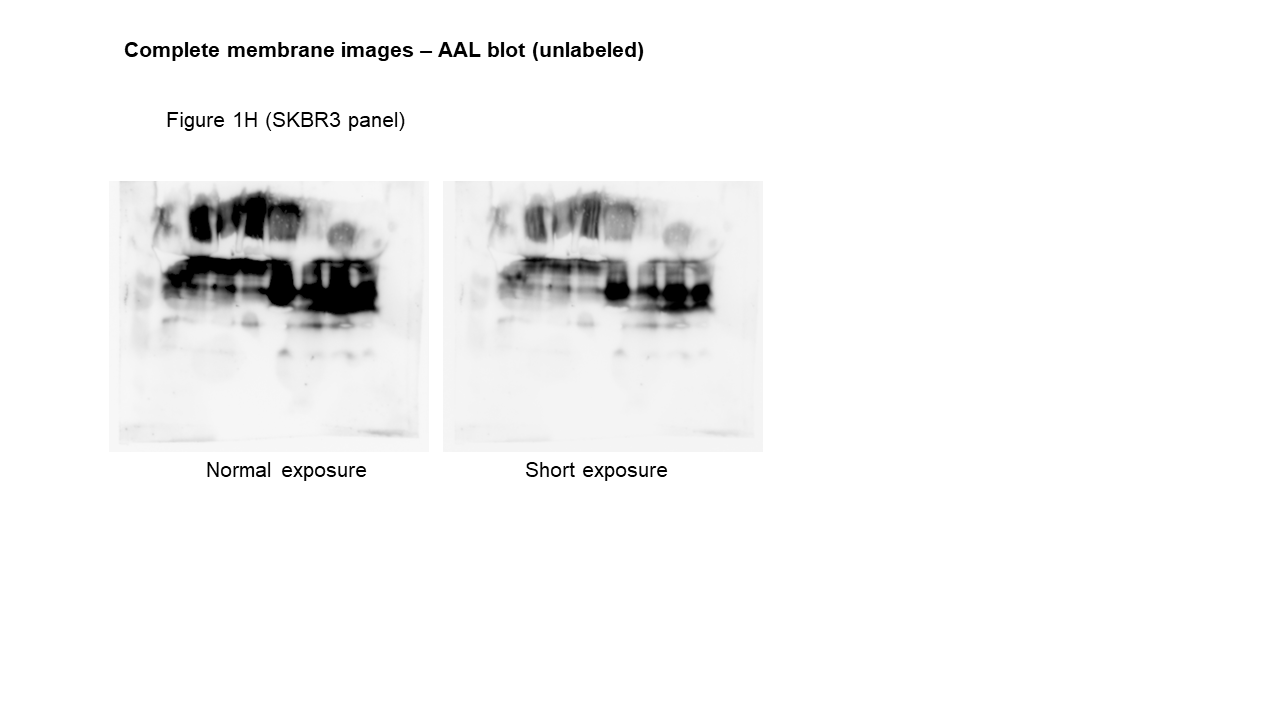

Supplement: Figure 1—source data 1. [file elife-75191-fig1-data1.zip › Figure 1/Figure 1H_5 (unlabeled).tif]

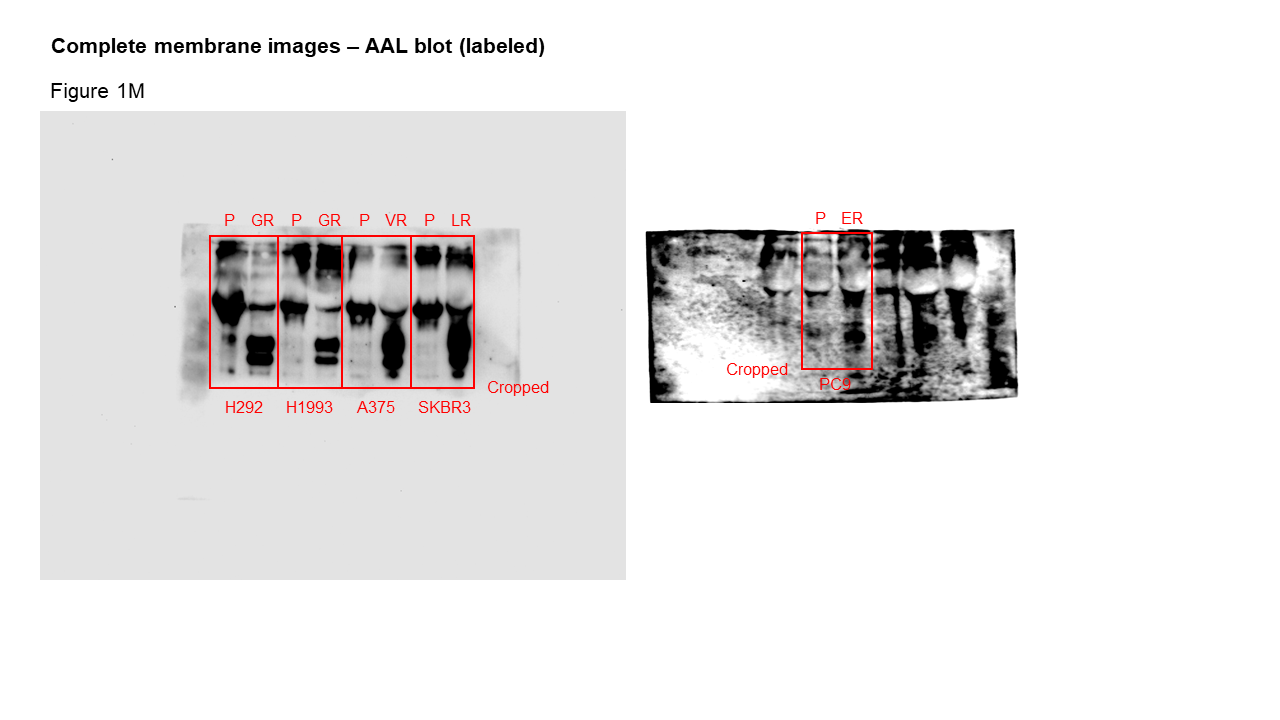

Supplement: Figure 1—source data 1. [file elife-75191-fig1-data1.zip › Figure 1/Figure 1M (labeled).tif]

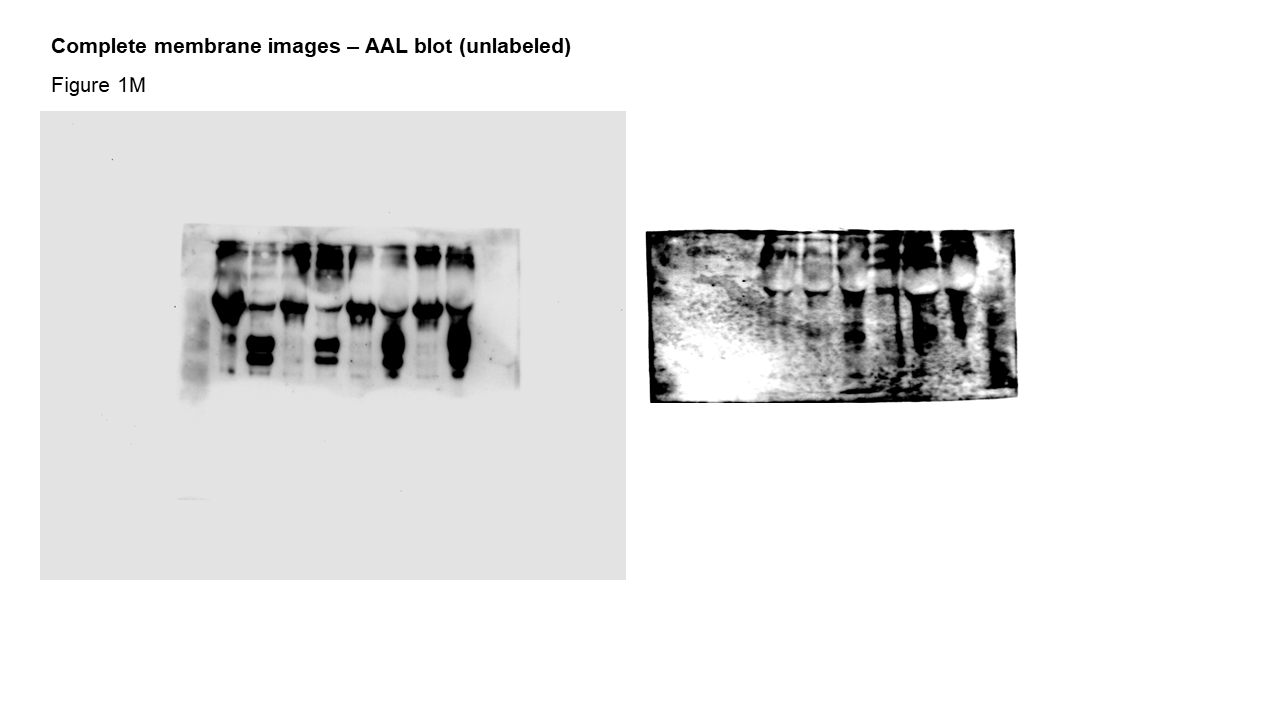

Supplement: Figure 1—source data 1. [file elife-75191-fig1-data1.zip › Figure 1/Figure 1M (unlabeled).tif]

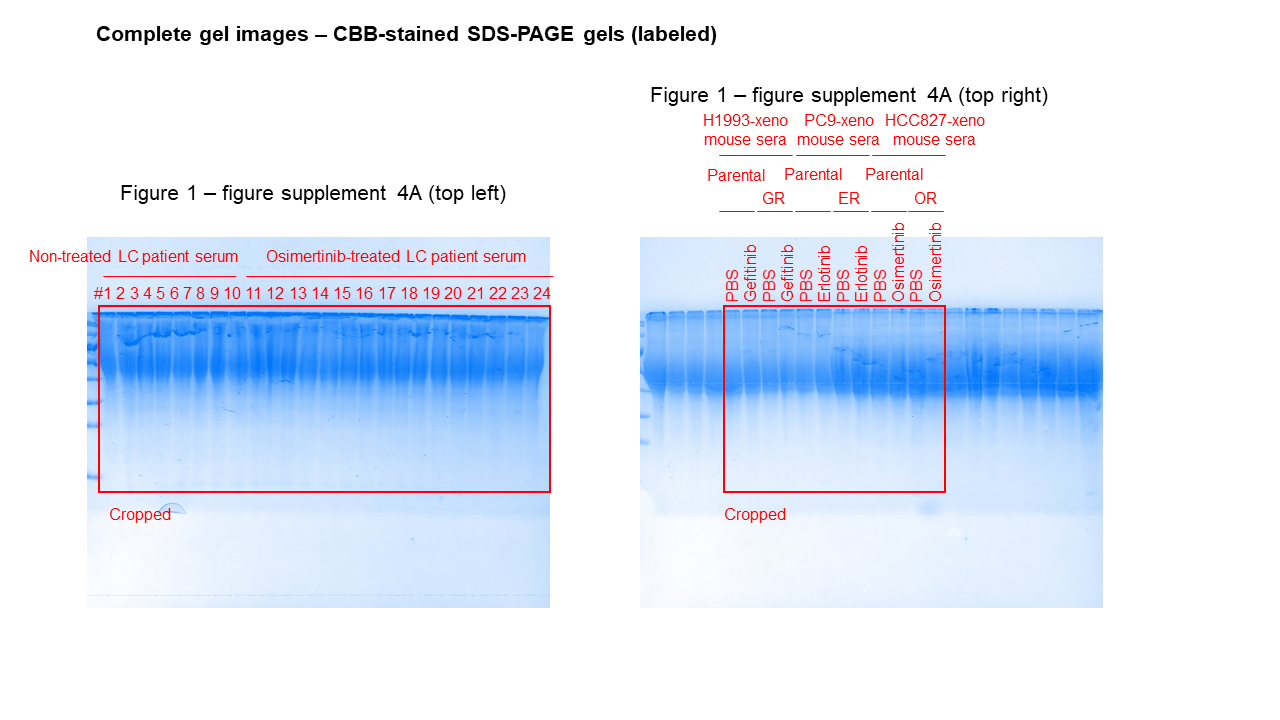

Supplement: Figure 1—source data 2. [file elife-75191-fig1-data2.zip › Figure 1-FS4/Figure 1_FS4A_1 (labeled).tif]

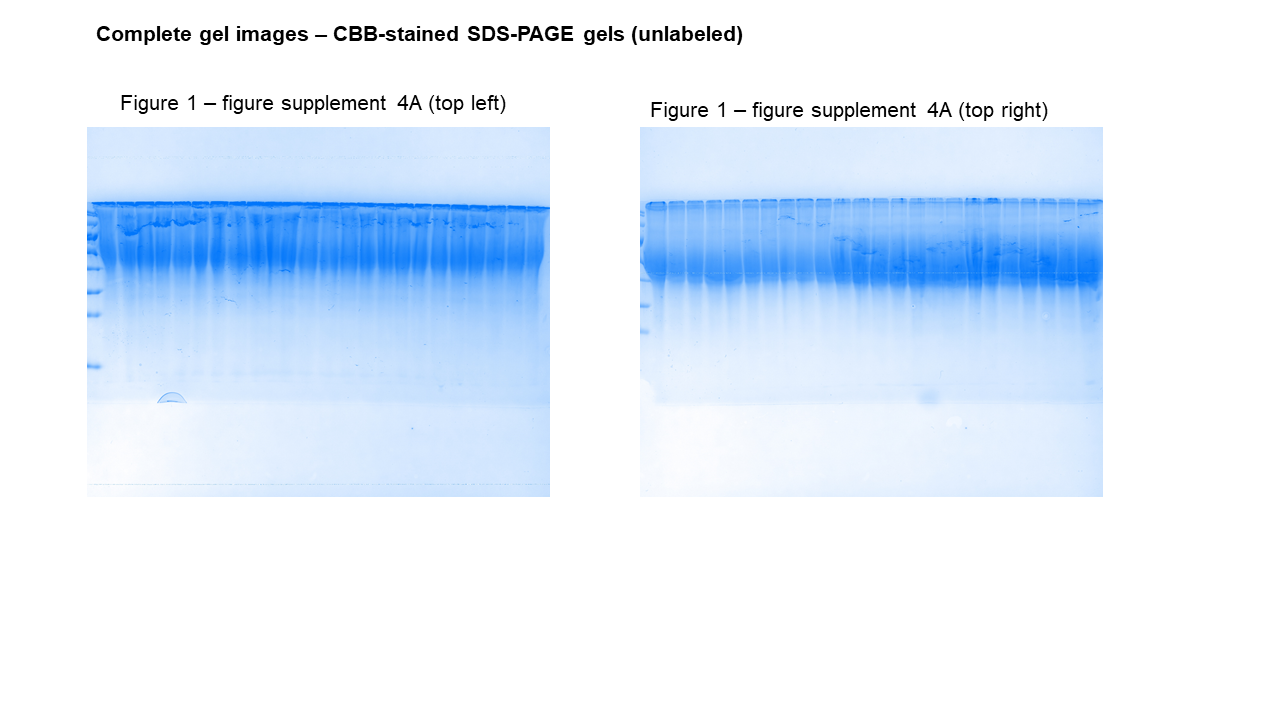

Supplement: Figure 1—source data 2. [file elife-75191-fig1-data2.zip › Figure 1-FS4/Figure 1_FS4A_1 (unlabeled).tif]

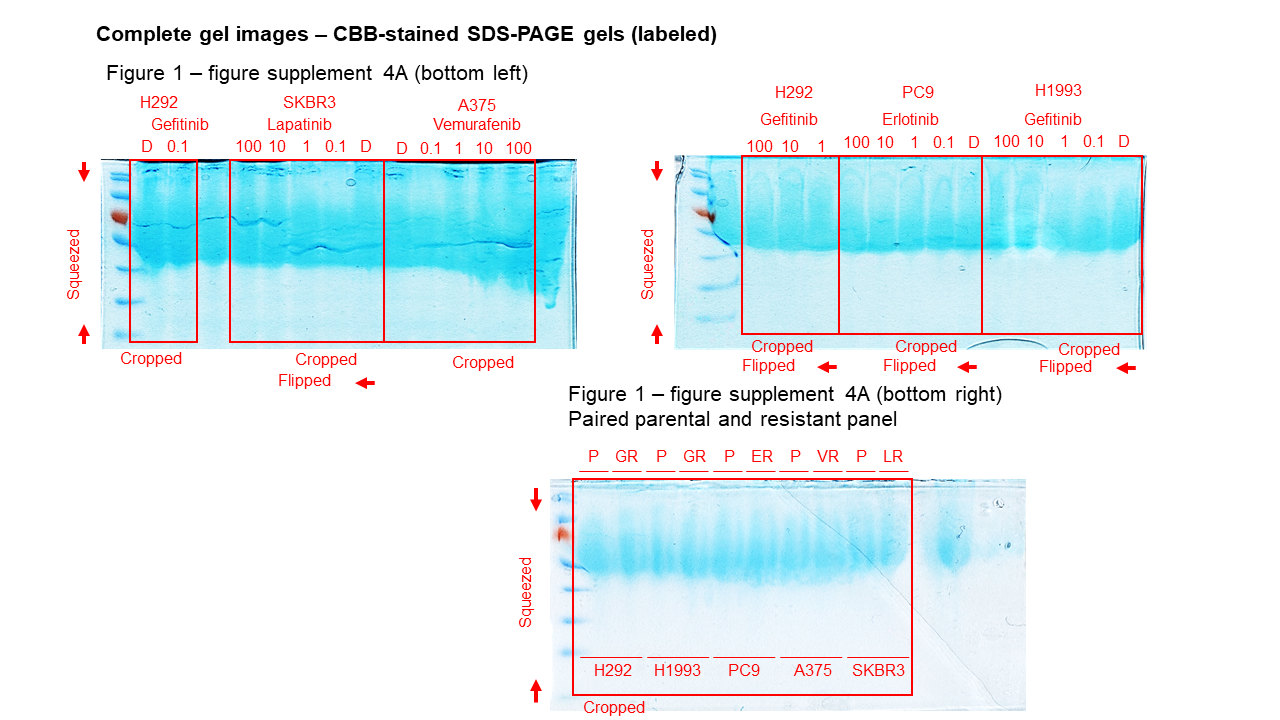

Supplement: Figure 1—source data 2. [file elife-75191-fig1-data2.zip › Figure 1-FS4/Figure 1_FS4A_2 (labeled).tif]

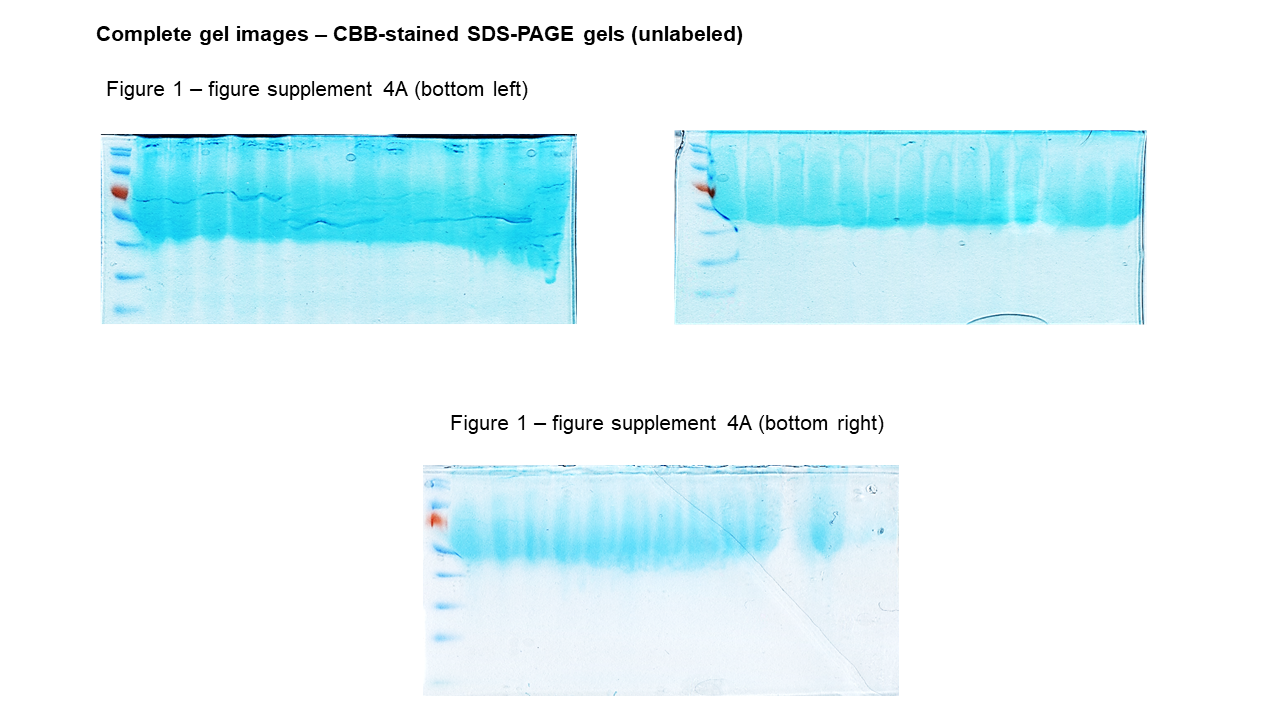

Supplement: Figure 1—source data 2. [file elife-75191-fig1-data2.zip › Figure 1-FS4/Figure 1_FS4A_2 (unlabeled).tif]

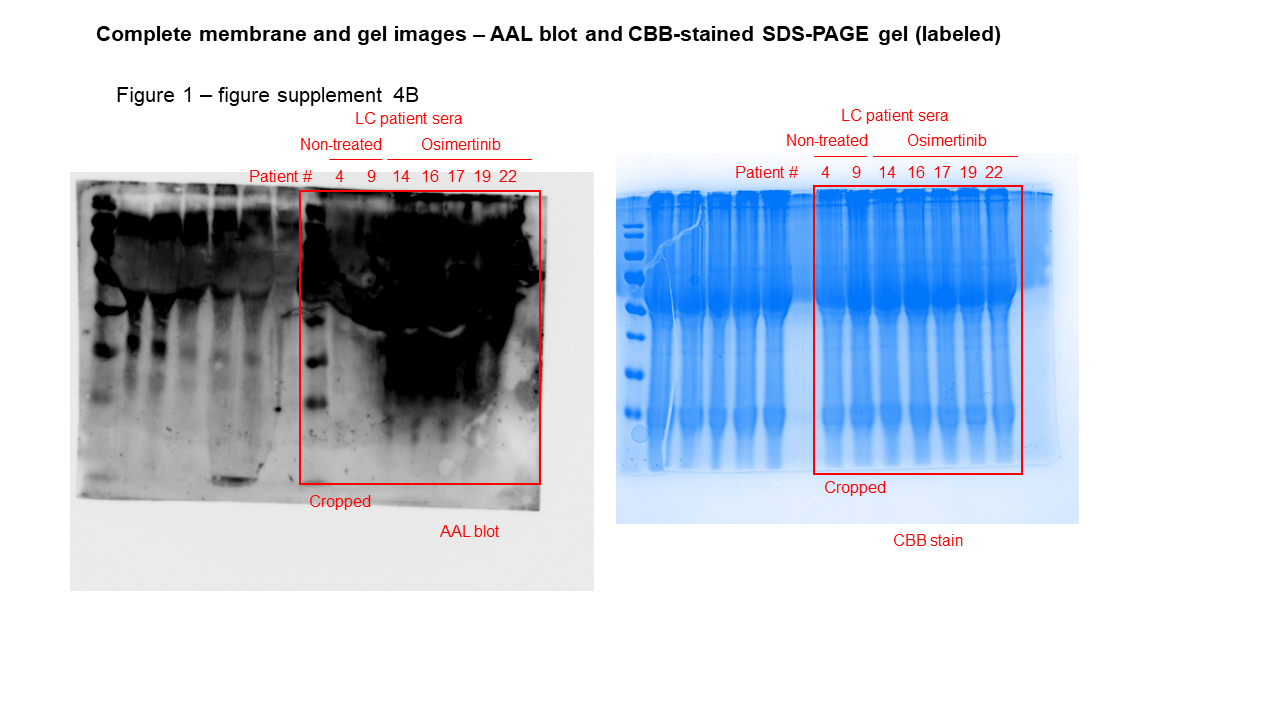

Supplement: Figure 1—source data 2. [file elife-75191-fig1-data2.zip › Figure 1-FS4/Figure 1_FS4B (labeled).tif]

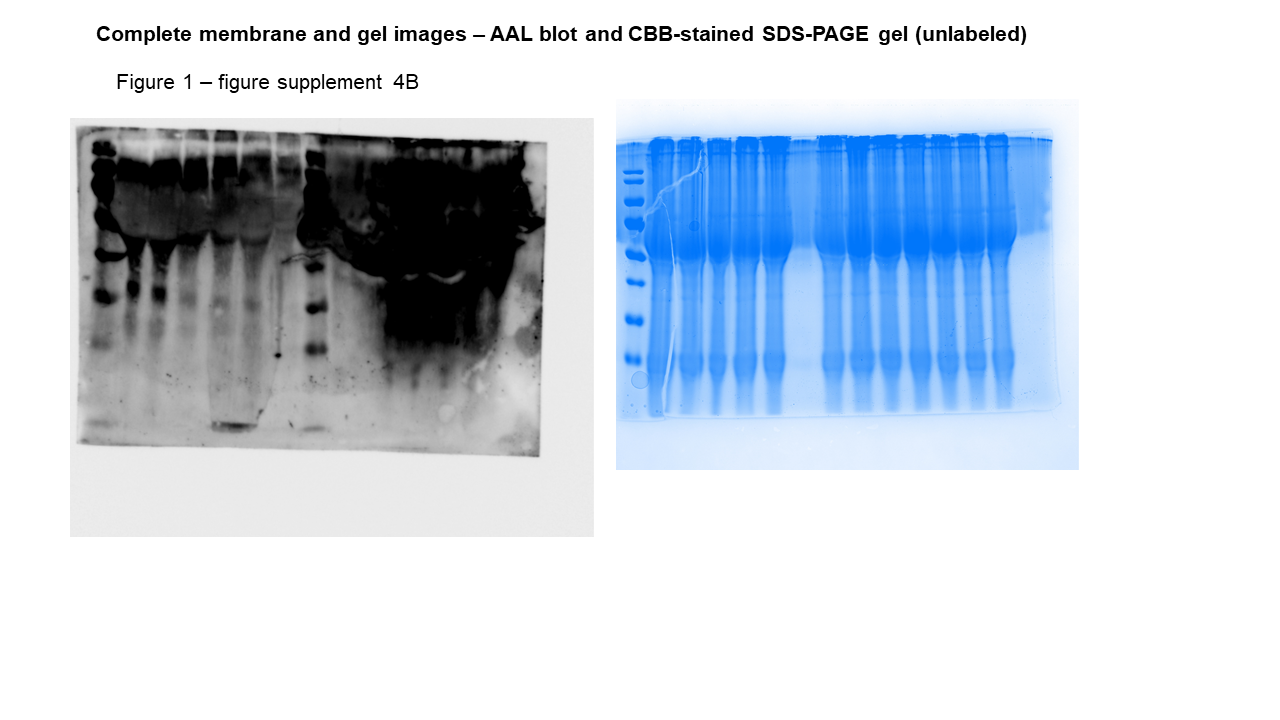

Supplement: Figure 1—source data 2. [file elife-75191-fig1-data2.zip › Figure 1-FS4/Figure 1_FS4B (unlabeled).tif]

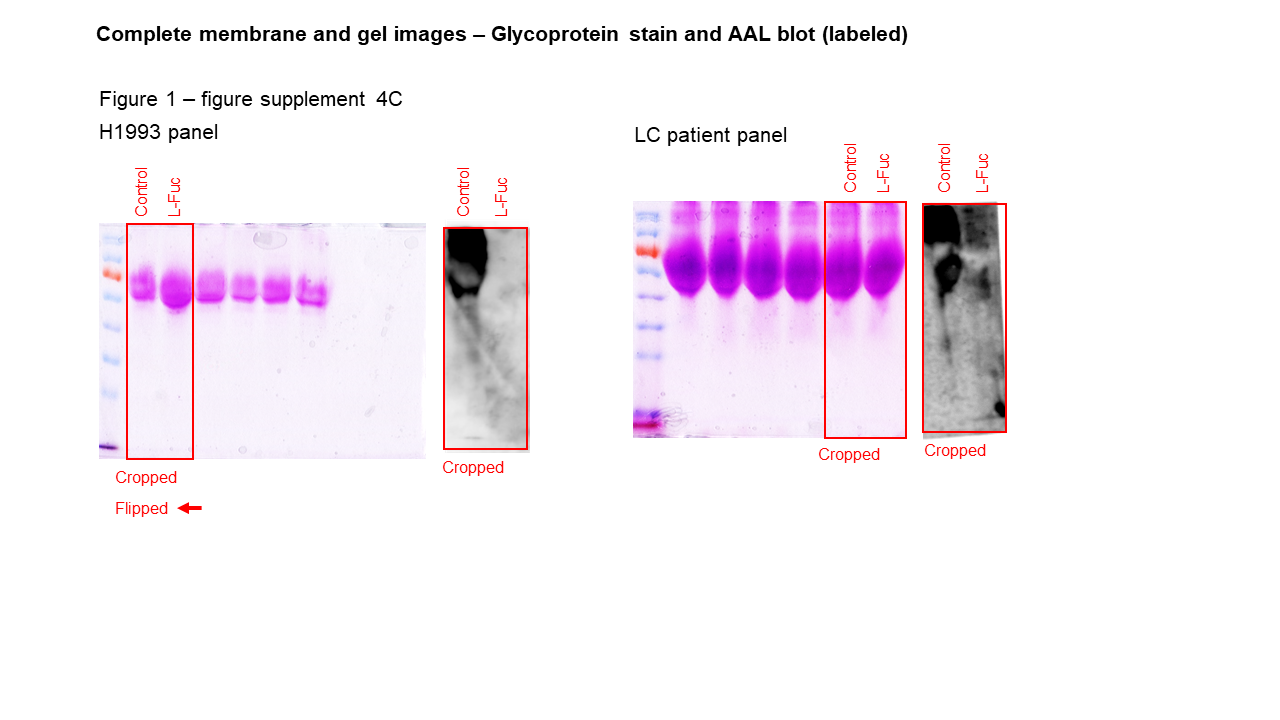

Supplement: Figure 1—source data 2. [file elife-75191-fig1-data2.zip › Figure 1-FS4/Figure 1_FS4C (labeled).tif]

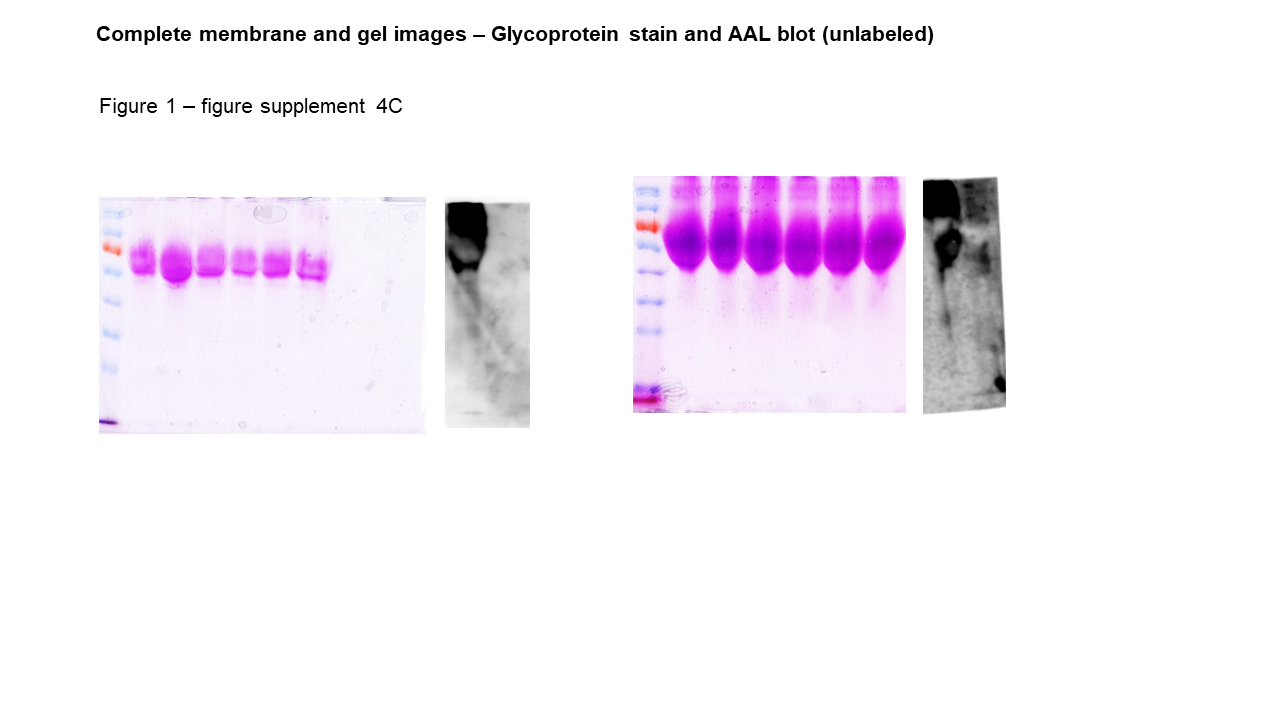

Supplement: Figure 1—source data 2. [file elife-75191-fig1-data2.zip › Figure 1-FS4/Figure 1_FS4C (unlabeled).tif]

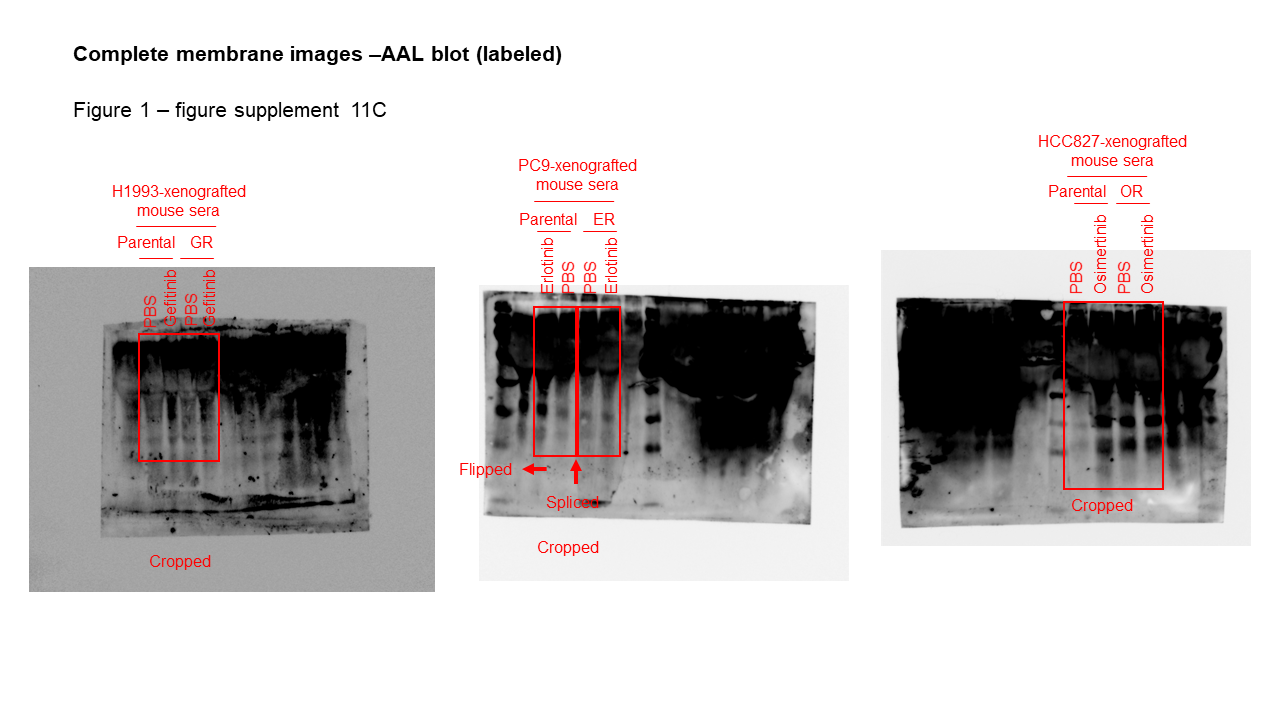

Supplement: Figure 1—source data 3. [file elife-75191-fig1-data3.zip › Figure 1-FS11/Figure 1_FS11C (labeled).tif]

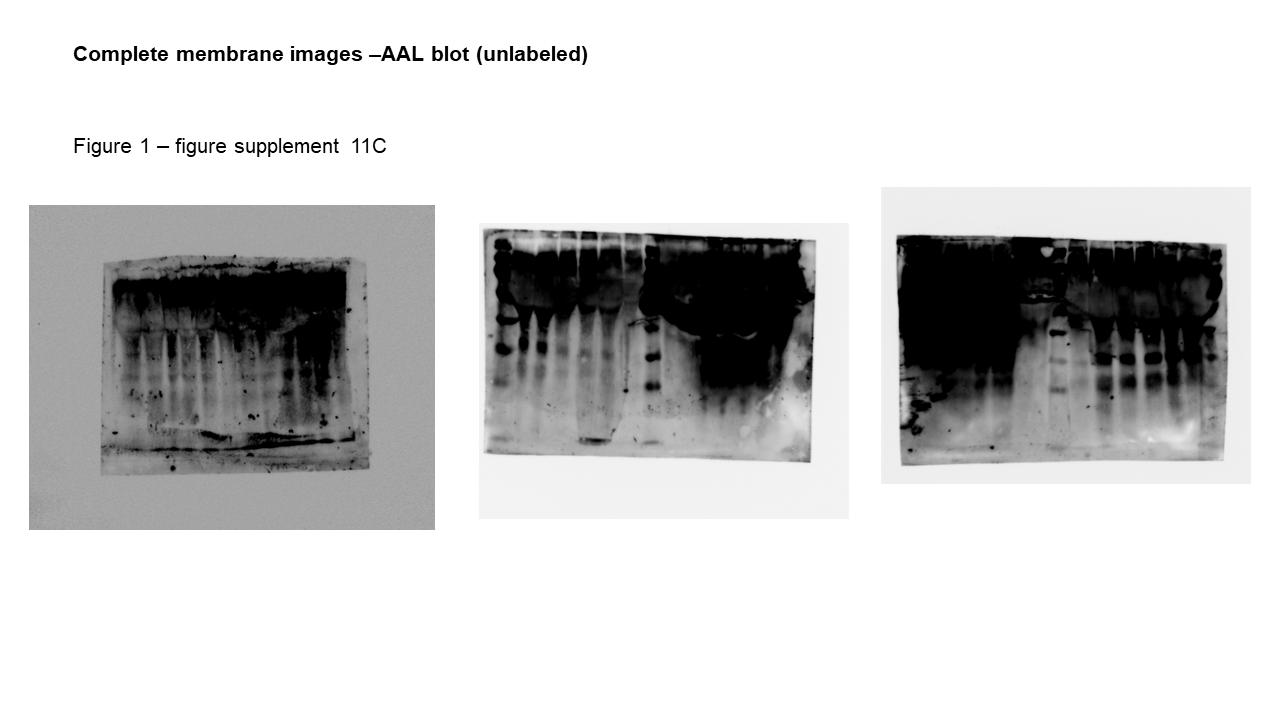

Supplement: Figure 1—source data 3. [file elife-75191-fig1-data3.zip › Figure 1-FS11/Figure 1_FS11C (unlabeled).tif]

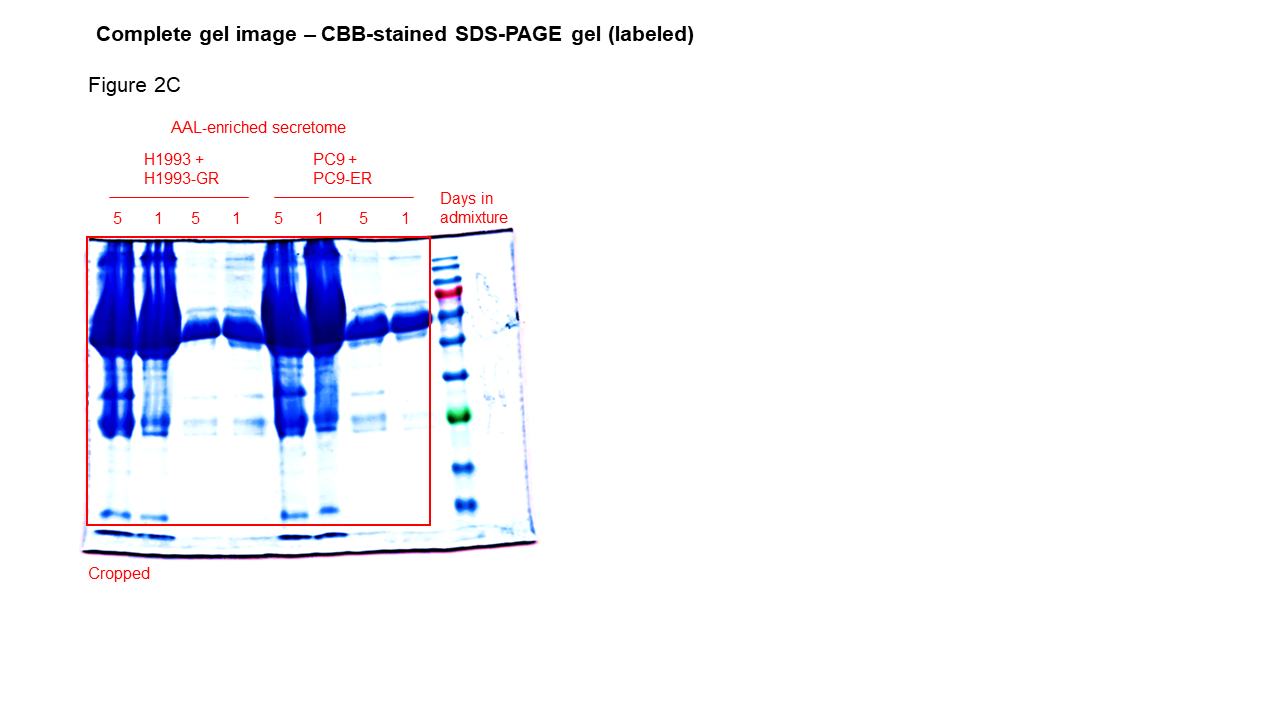

Supplement: Figure 2—source data 1. [file elife-75191-fig2-data1.zip › Figure 2/Figure 2C (labeled).tif]

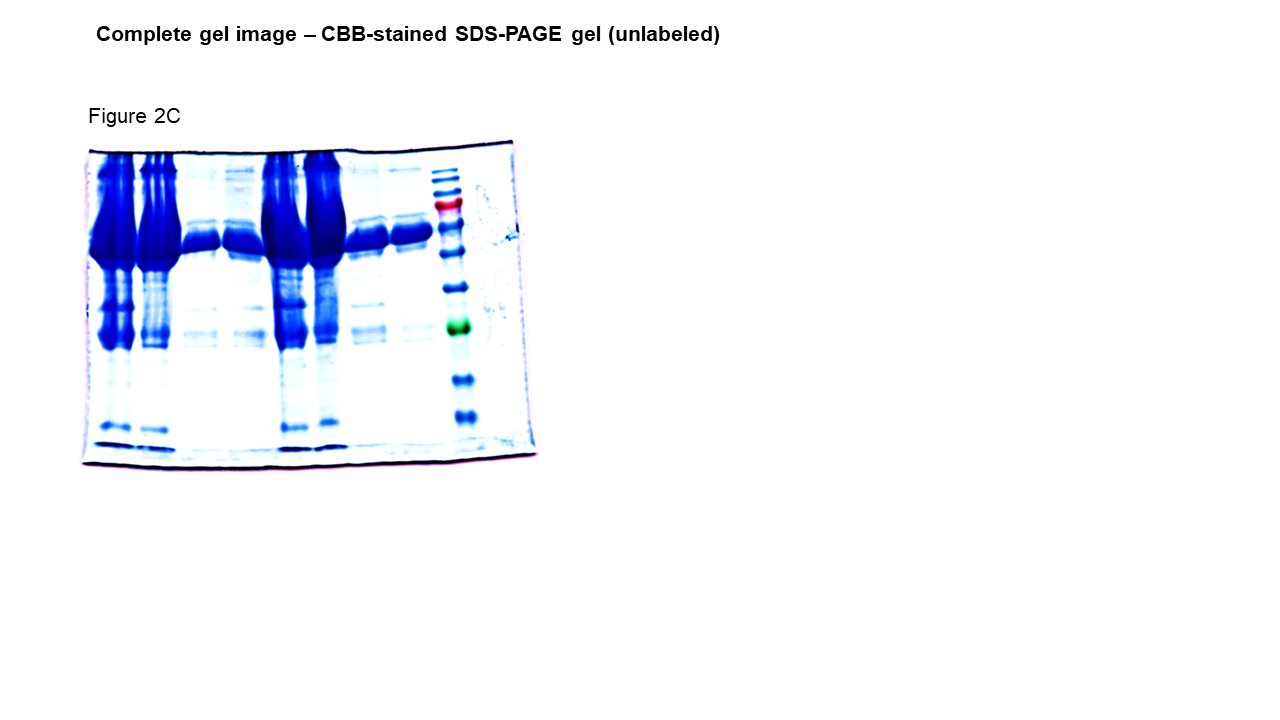

Supplement: Figure 2—source data 1. [file elife-75191-fig2-data1.zip › Figure 2/Figure 2C (unlabeled).tif]

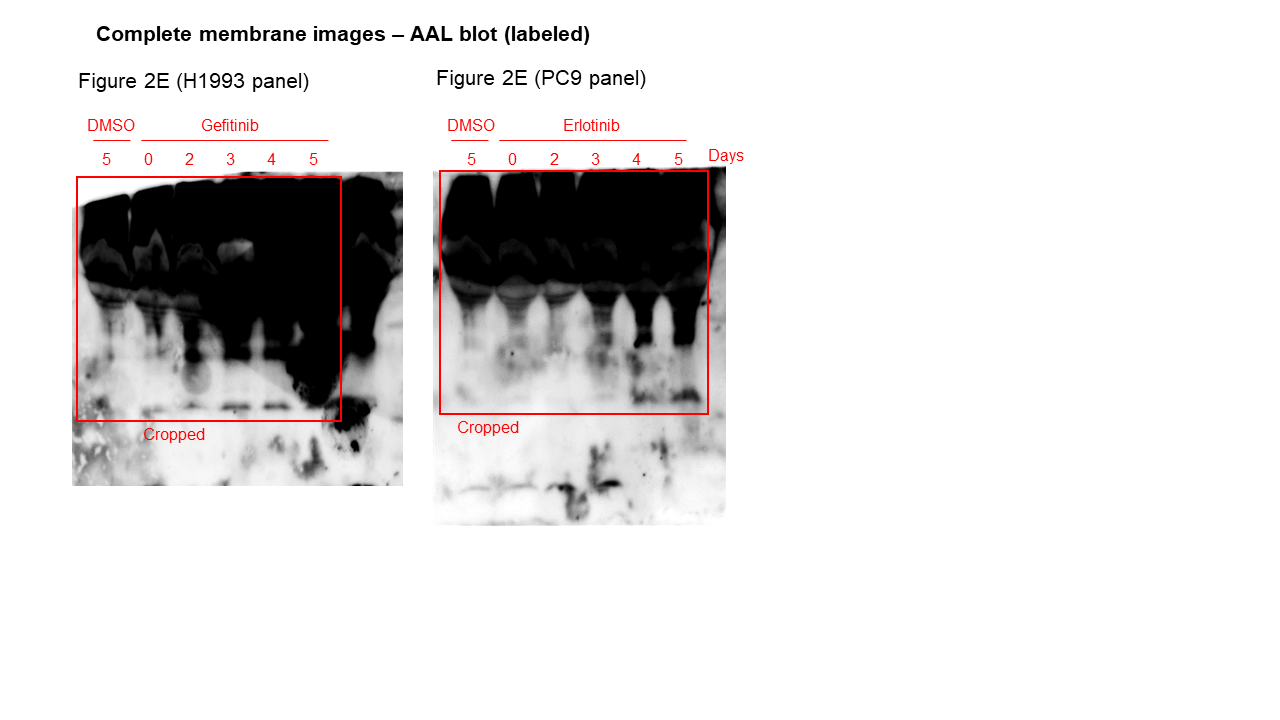

Supplement: Figure 2—source data 1. [file elife-75191-fig2-data1.zip › Figure 2/Figure 2E (labeled).tif]

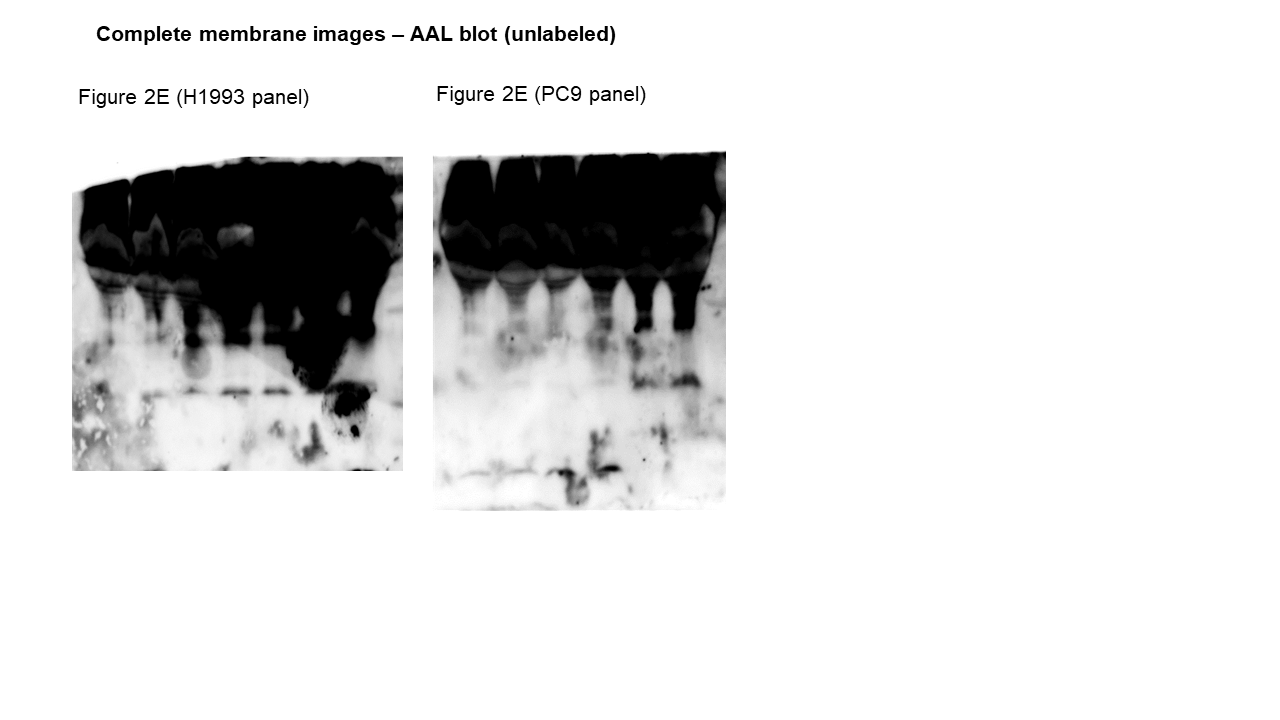

Supplement: Figure 2—source data 1. [file elife-75191-fig2-data1.zip › Figure 2/Figure 2E (unlabeled).tif]

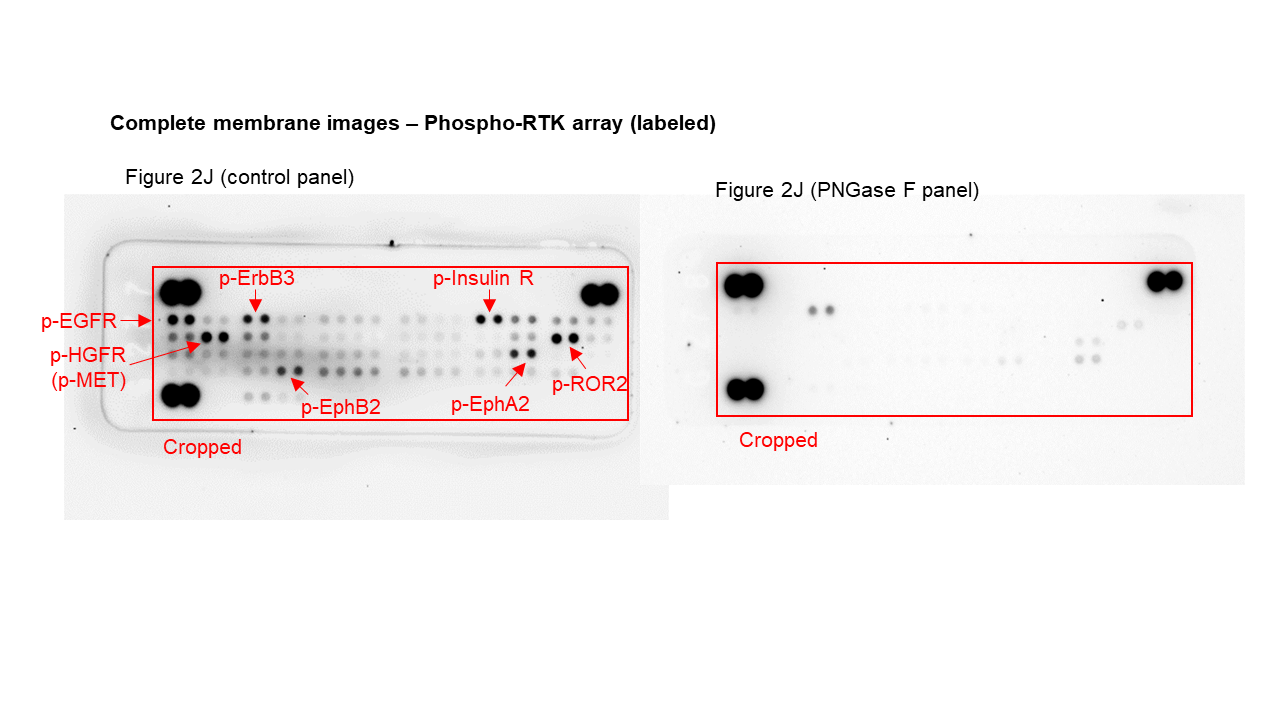

Supplement: Figure 2—source data 1. [file elife-75191-fig2-data1.zip › Figure 2/Figure 2J (labeled).tif]

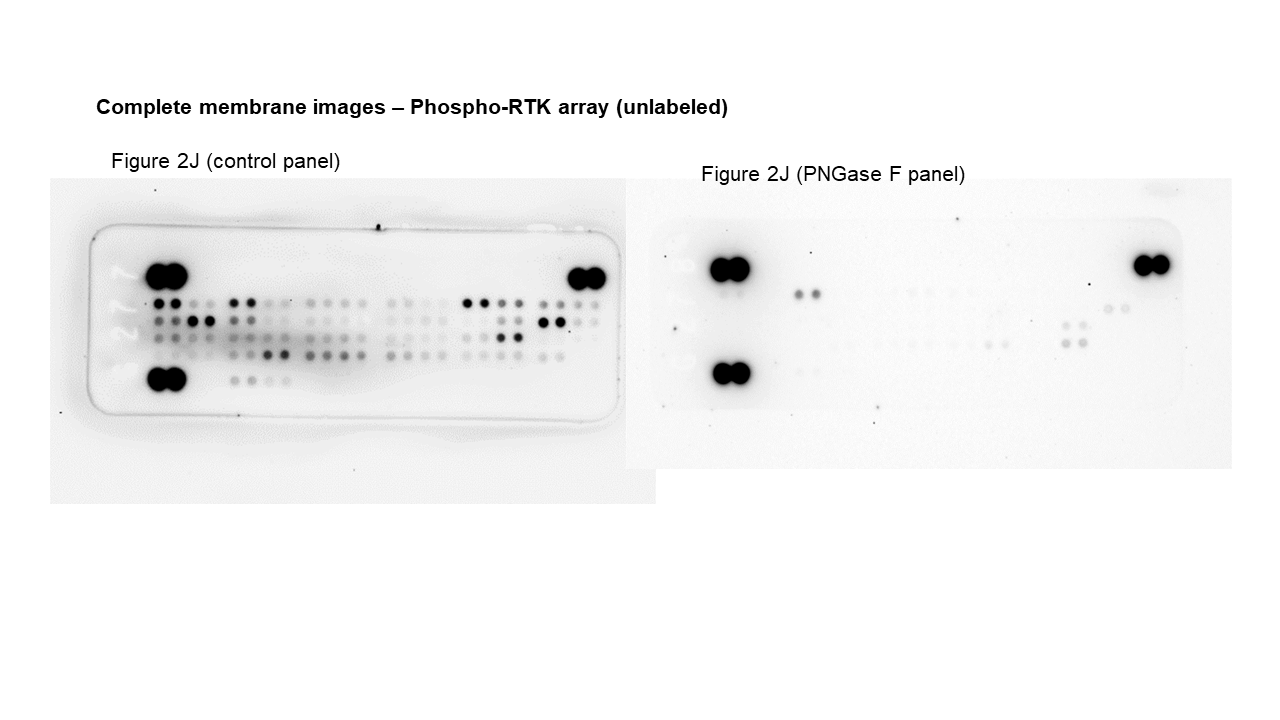

Supplement: Figure 2—source data 1. [file elife-75191-fig2-data1.zip › Figure 2/Figure 2J (unlabeled).tif]

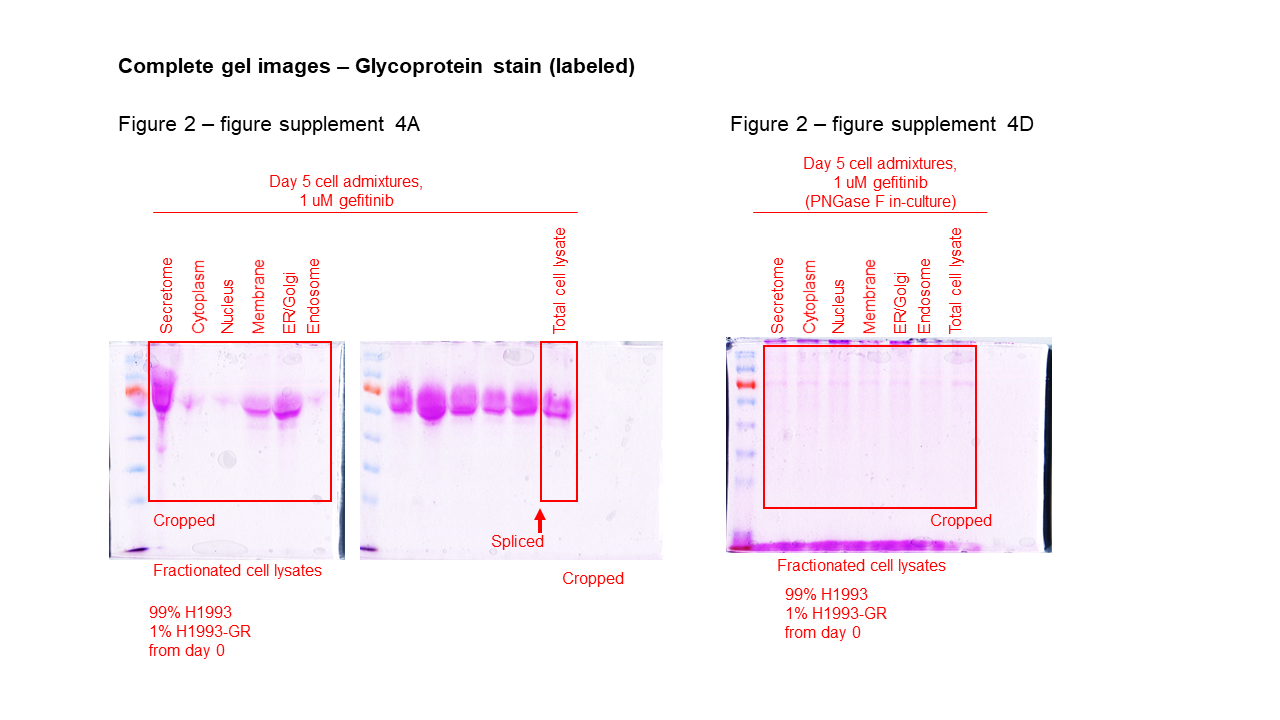

Supplement: Figure 2—source data 2. [file elife-75191-fig2-data2.zip › Figure 2-FS4/Figure 2_FS4 (labeled).tif]

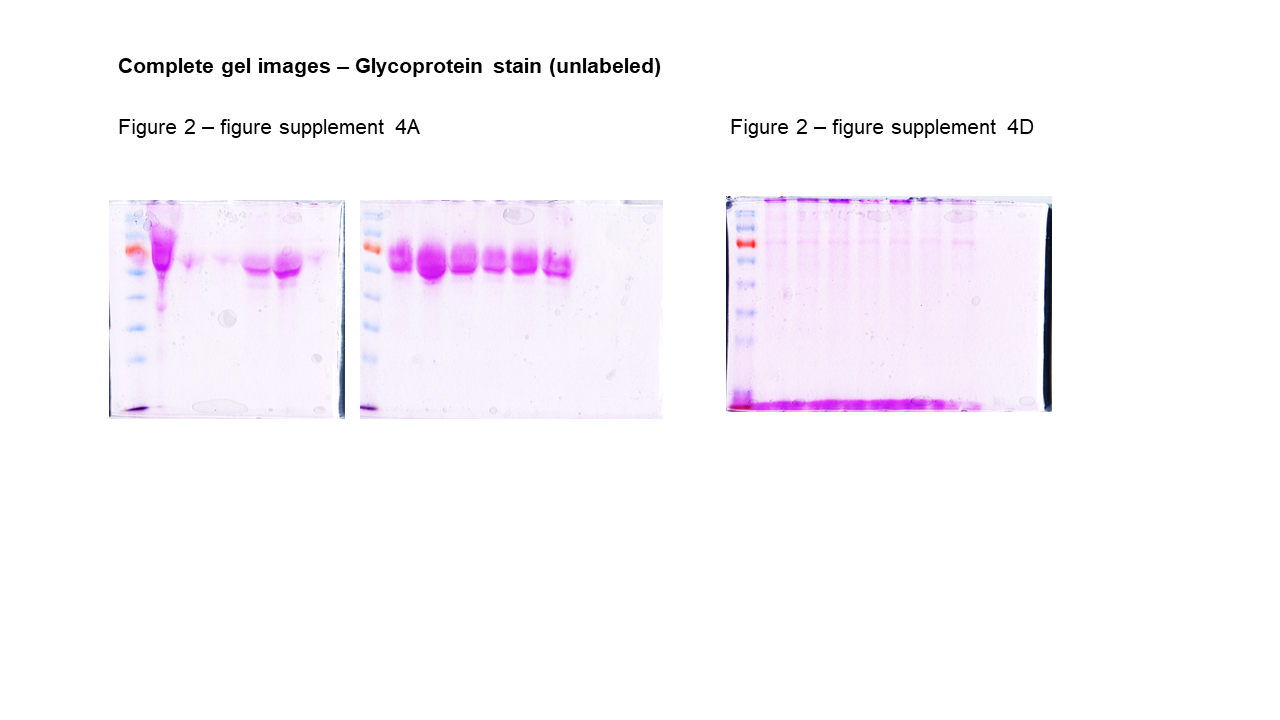

Supplement: Figure 2—source data 2. [file elife-75191-fig2-data2.zip › Figure 2-FS4/Figure 2_FS4 (unlabeled).tif]

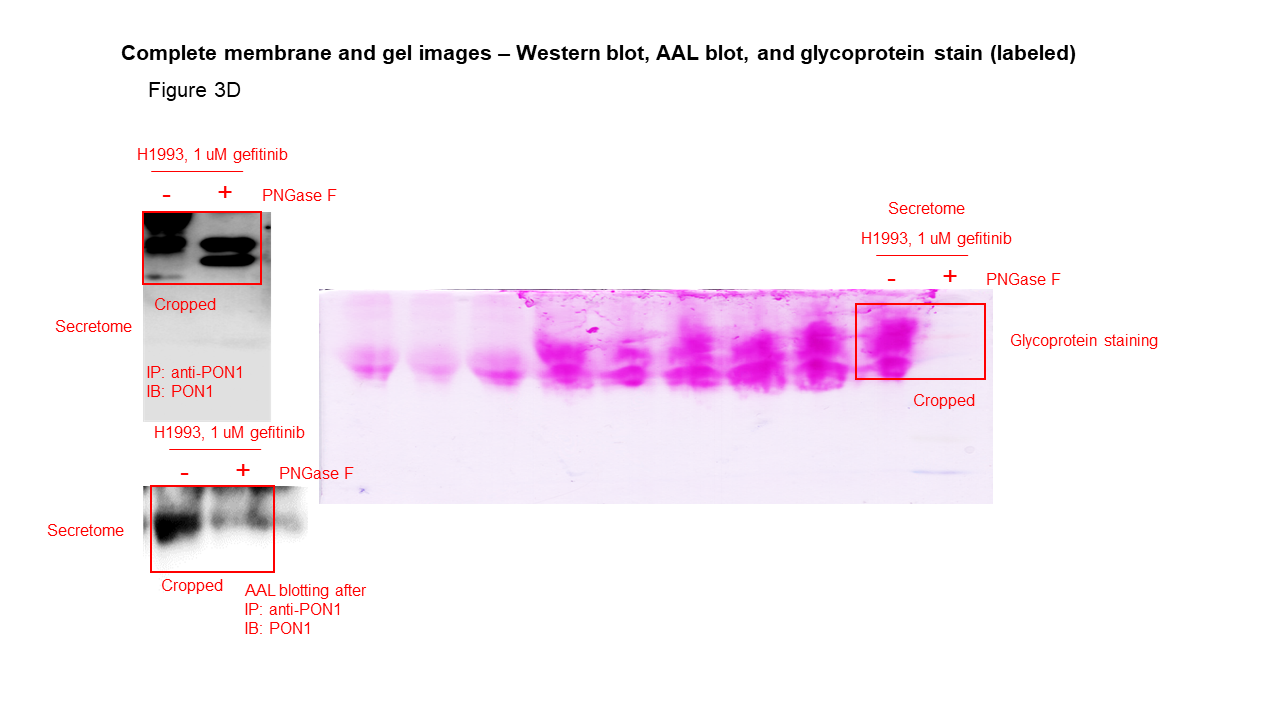

Supplement: Figure 3—source data 1. [file elife-75191-fig3-data1.zip › Figure 3/Figure 3D (labeled).tif]

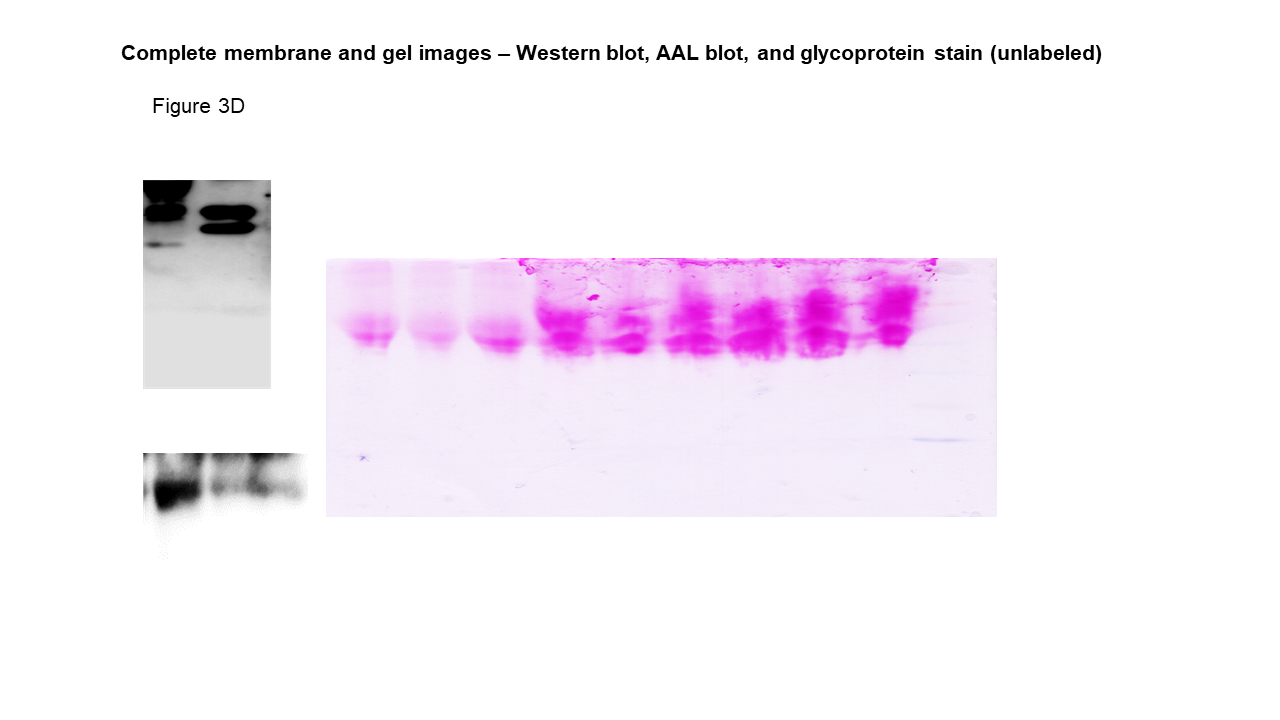

Supplement: Figure 3—source data 1. [file elife-75191-fig3-data1.zip › Figure 3/Figure 3D (unlabeled).tif]

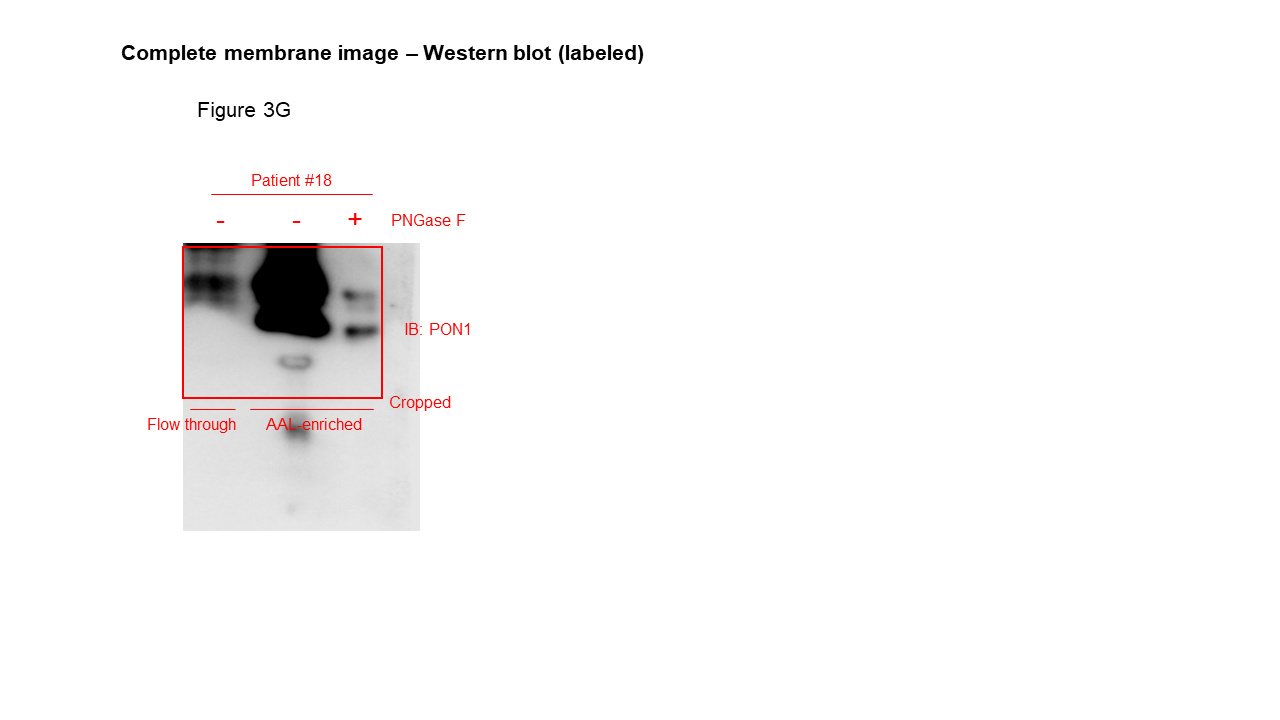

Supplement: Figure 3—source data 1. [file elife-75191-fig3-data1.zip › Figure 3/Figure 3G (labeled).tif]

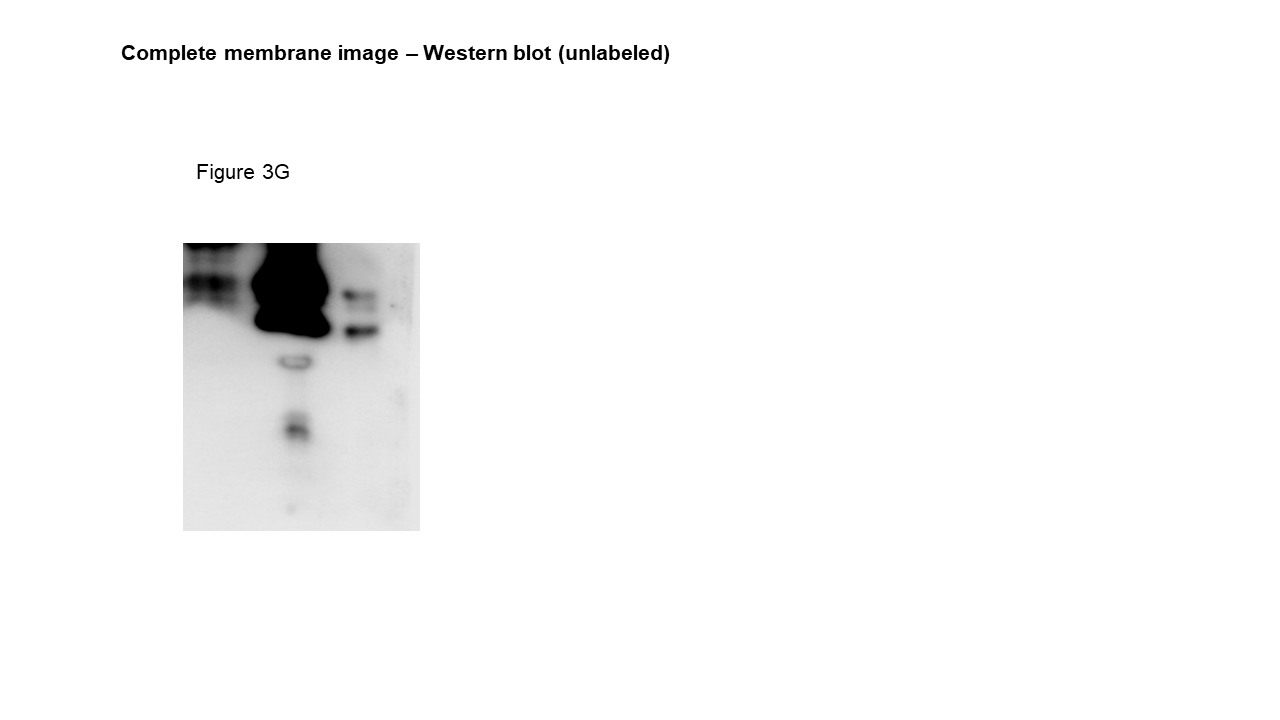

Supplement: Figure 3—source data 1. [file elife-75191-fig3-data1.zip › Figure 3/Figure 3G (unlabeled).tif]

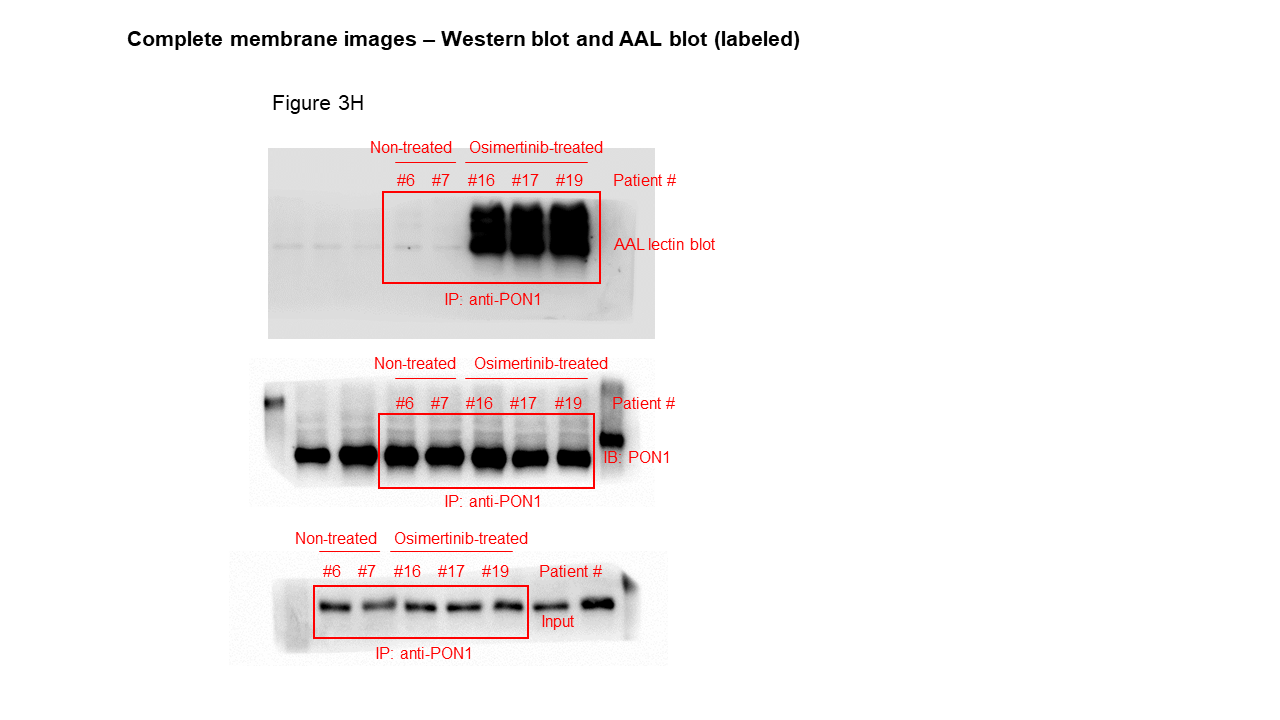

Supplement: Figure 3—source data 1. [file elife-75191-fig3-data1.zip › Figure 3/Figure 3H (labeled).tif]

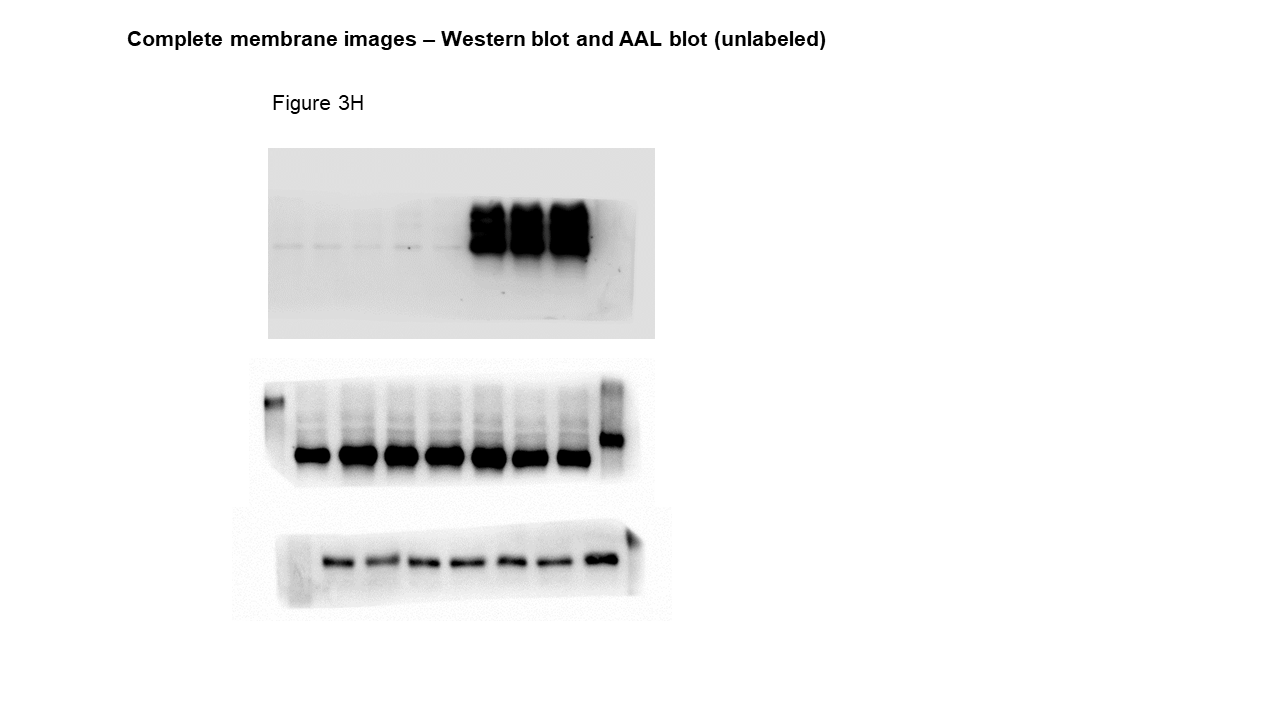

Supplement: Figure 3—source data 1. [file elife-75191-fig3-data1.zip › Figure 3/Figure 3H (unlabeled).tif]

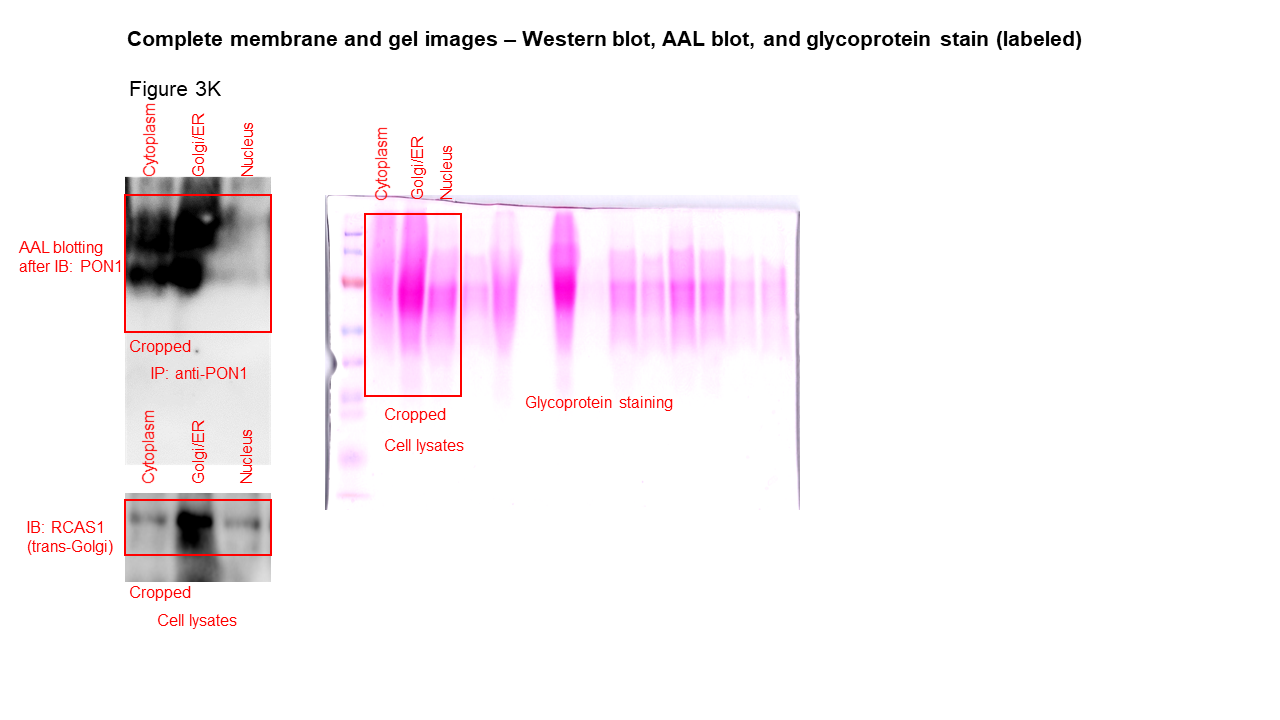

Supplement: Figure 3—source data 1. [file elife-75191-fig3-data1.zip › Figure 3/Figure 3K (labeled).tif]

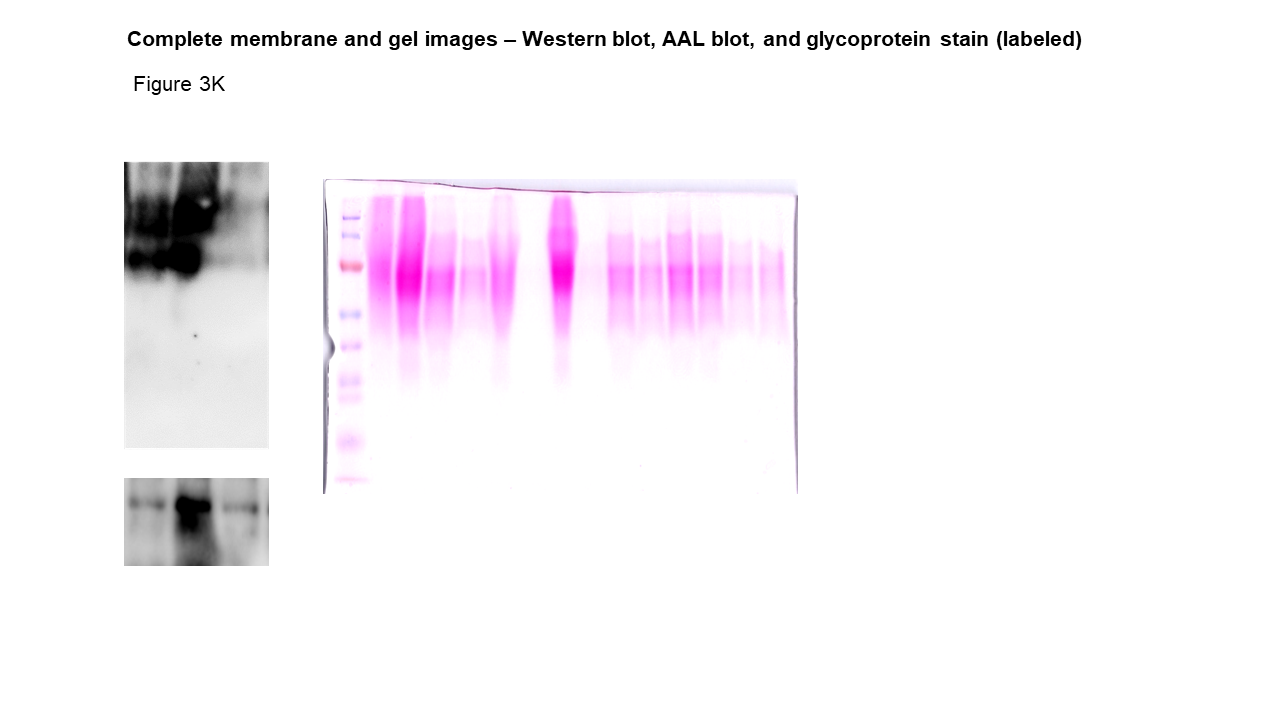

Supplement: Figure 3—source data 1. [file elife-75191-fig3-data1.zip › Figure 3/Figure 3K (unlabeled).tif]

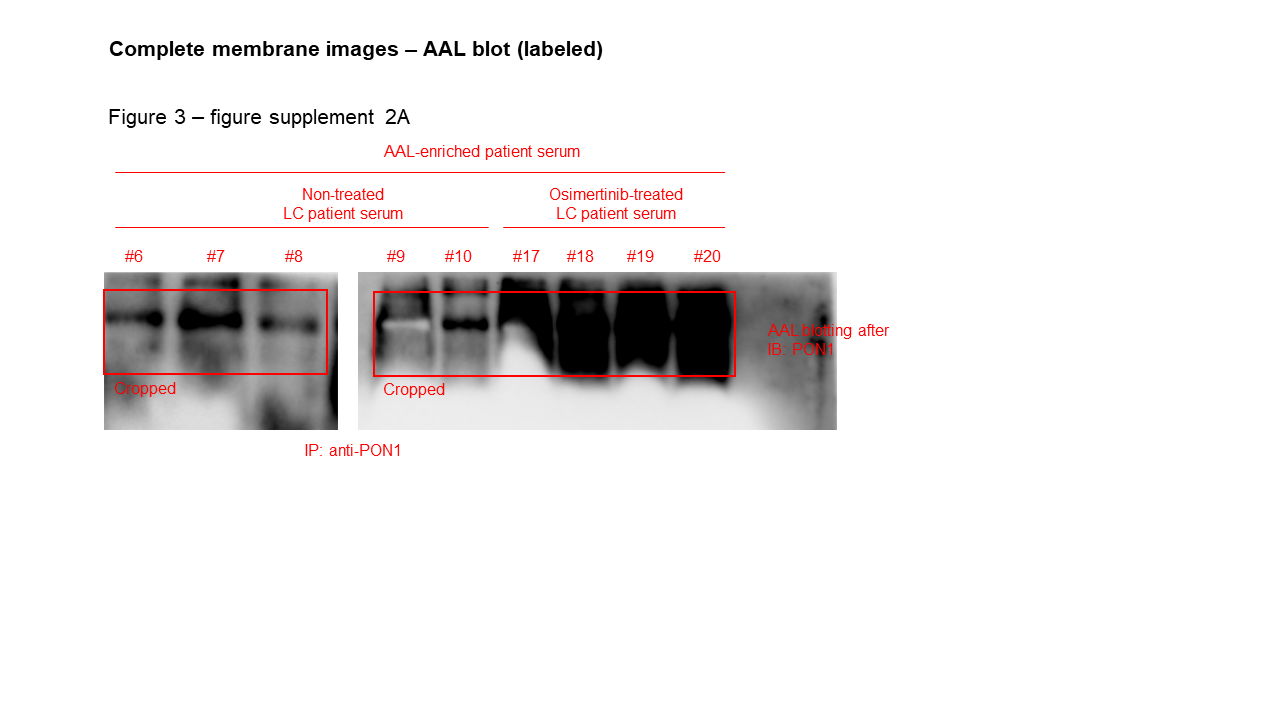

Supplement: Figure 3—source data 2. [file elife-75191-fig3-data2.zip › Figure 3-FS2/Figure 3_FS2A (labeled).tif]

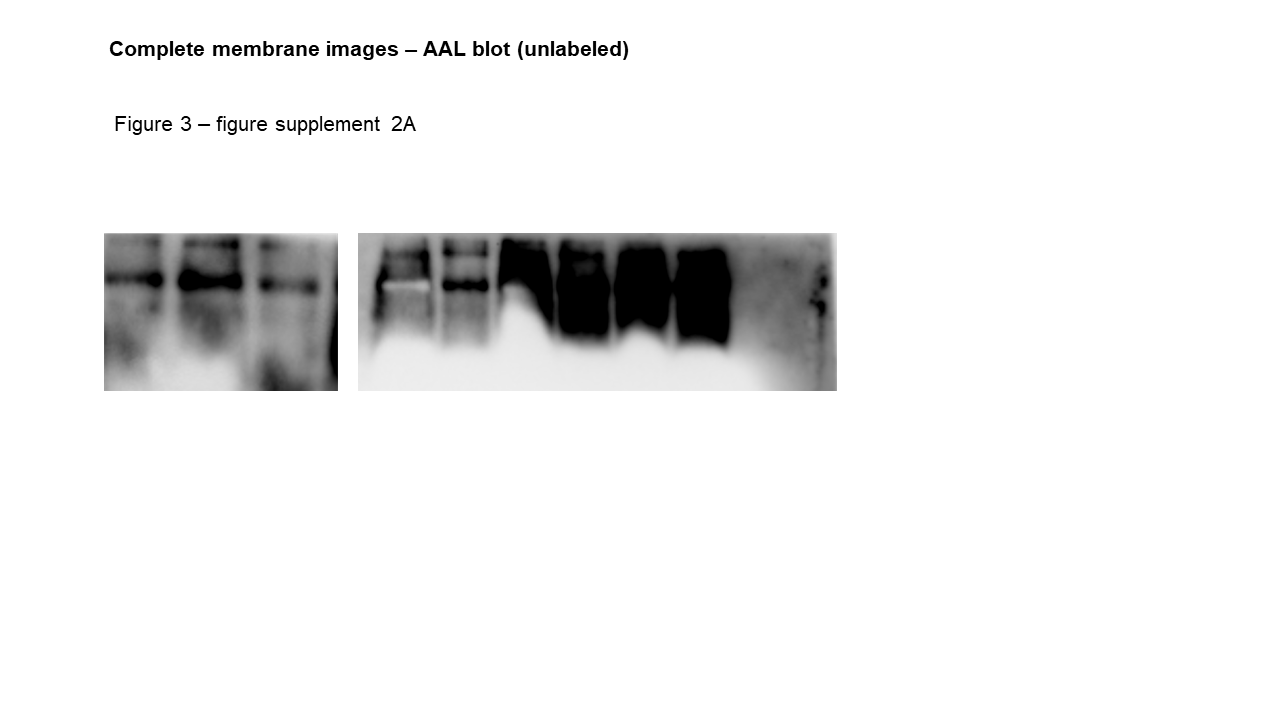

Supplement: Figure 3—source data 2. [file elife-75191-fig3-data2.zip › Figure 3-FS2/Figure 3_FS2A (unlabeled).tif]

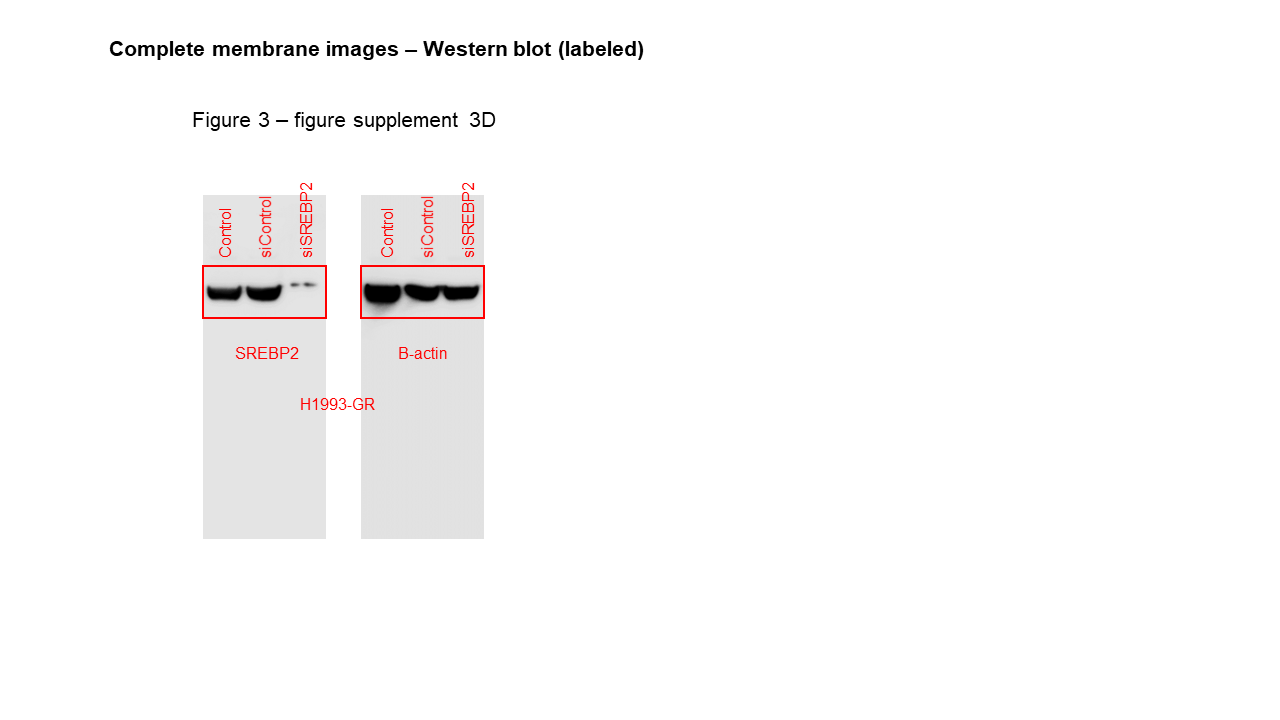

Supplement: Figure 3—source data 3. [file elife-75191-fig3-data3.zip › Figure 3-FS3/Figure 3_FS3D (labeled).tif]

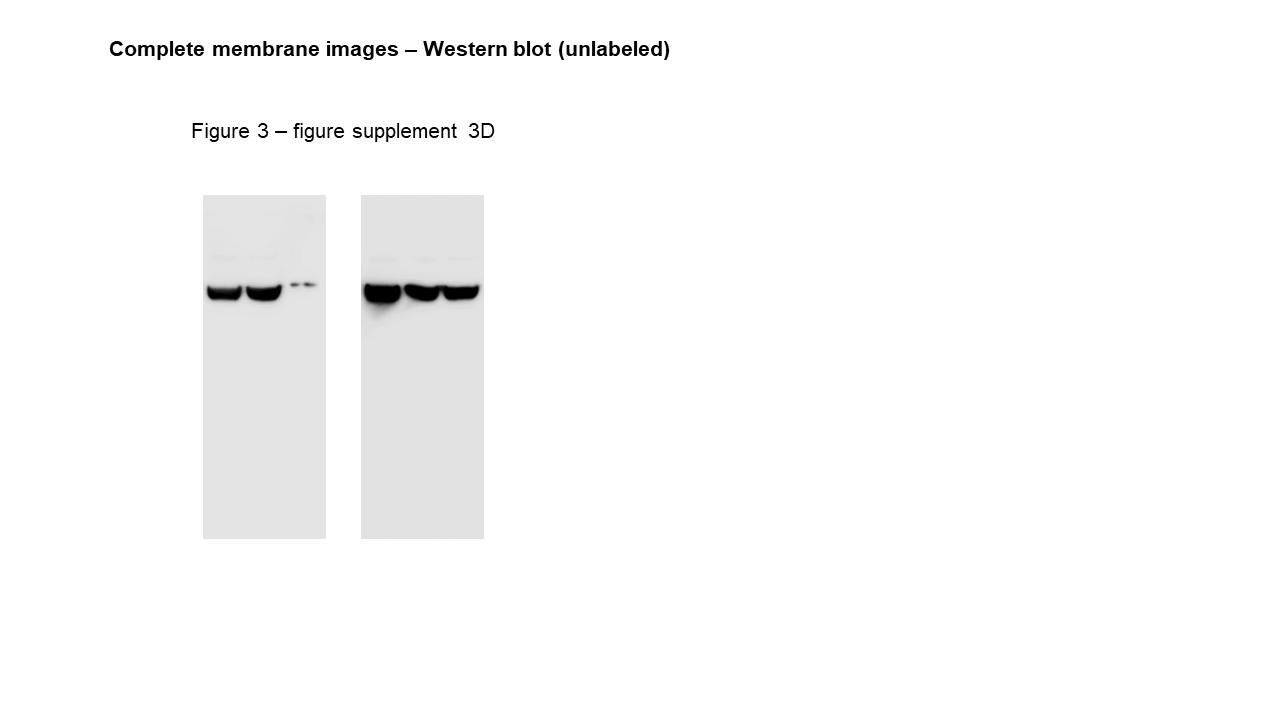

Supplement: Figure 3—source data 3. [file elife-75191-fig3-data3.zip › Figure 3-FS3/Figure 3_FS3D (unlabeled).tif]

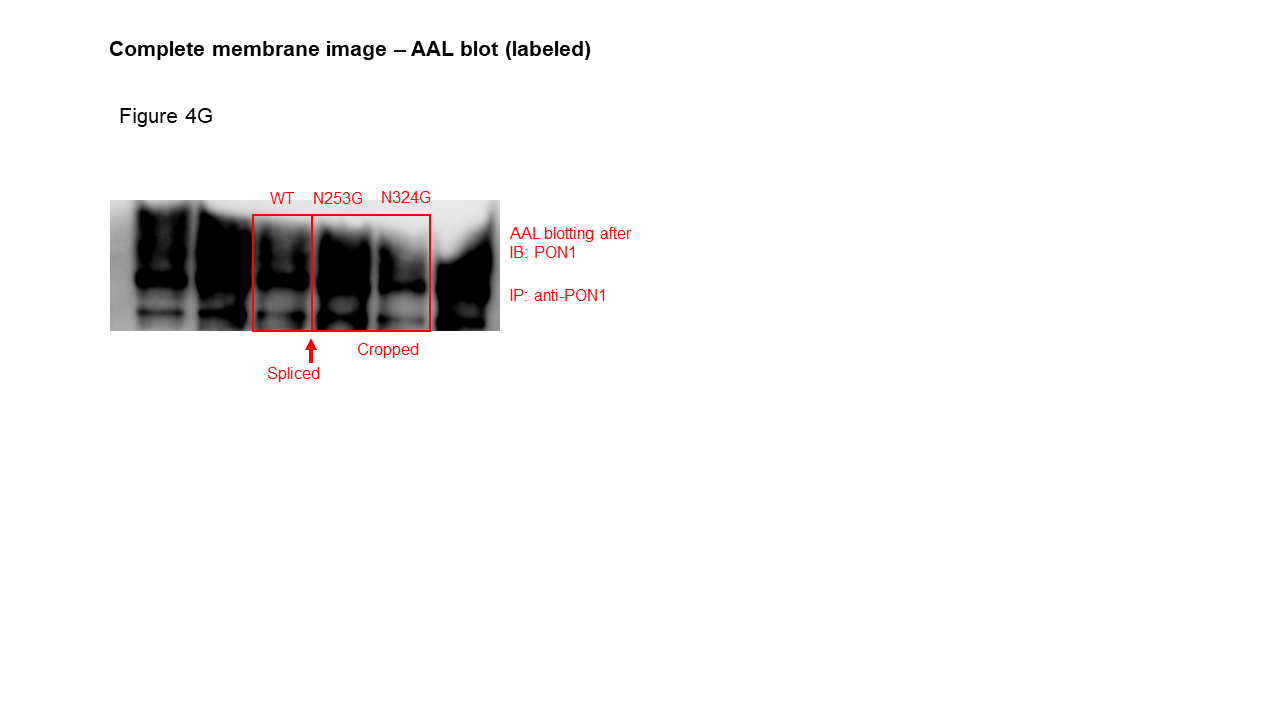

Supplement: Figure 4—source data 1. [file elife-75191-fig4-data1.zip › Figure 4/Figure 4G (labeled).tif]

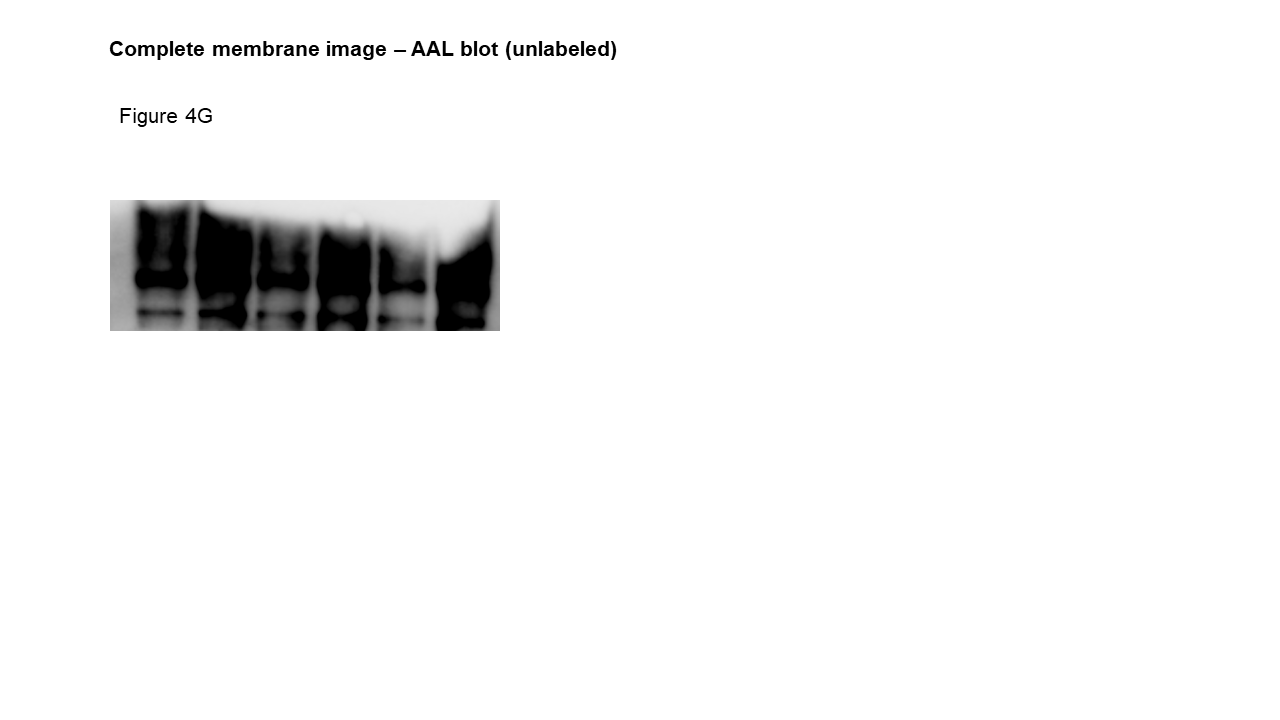

Supplement: Figure 4—source data 1. [file elife-75191-fig4-data1.zip › Figure 4/Figure 4G (unlabeled).tif]

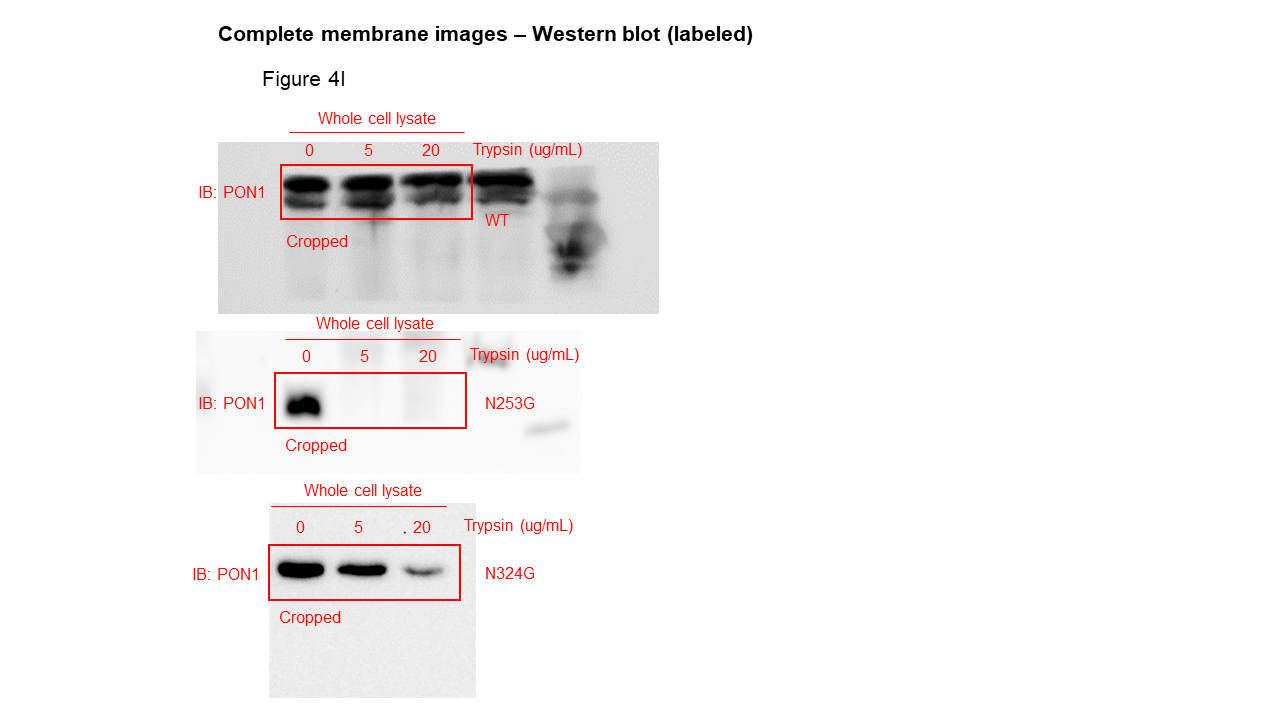

Supplement: Figure 4—source data 1. [file elife-75191-fig4-data1.zip › Figure 4/Figure 4I (labeled).tif]

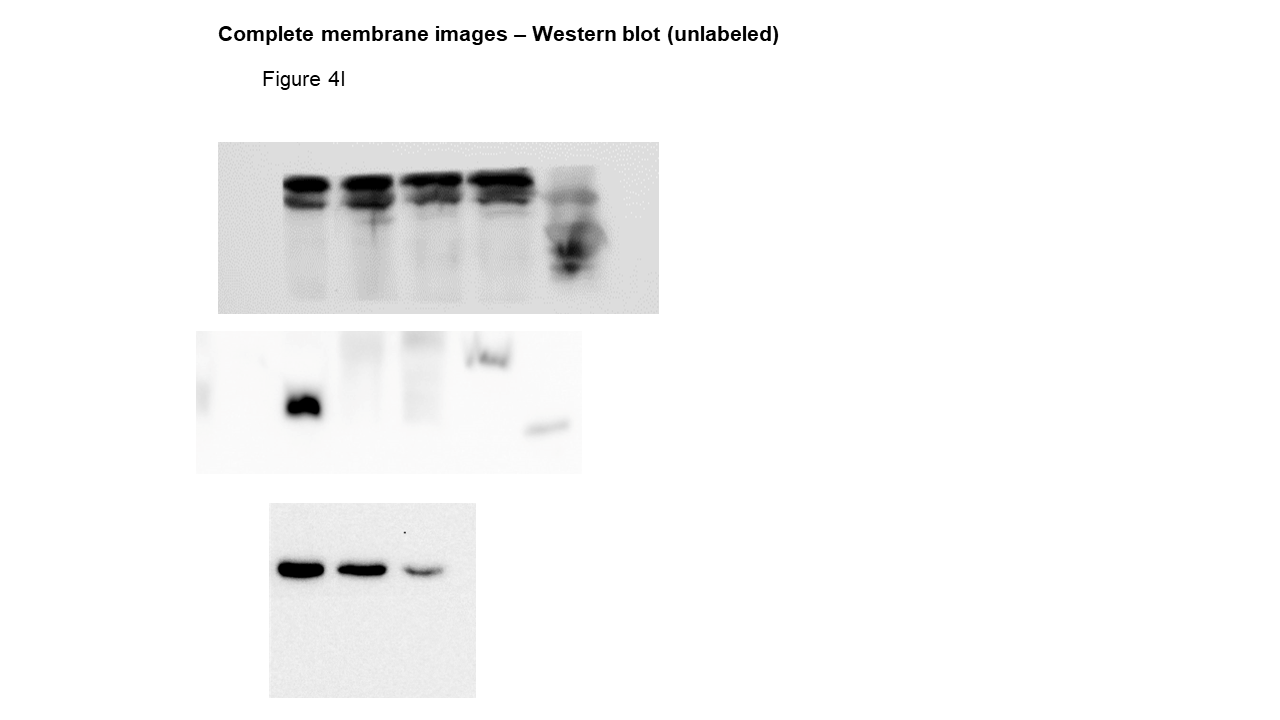

Supplement: Figure 4—source data 1. [file elife-75191-fig4-data1.zip › Figure 4/Figure 4I (unlabeled).tif]

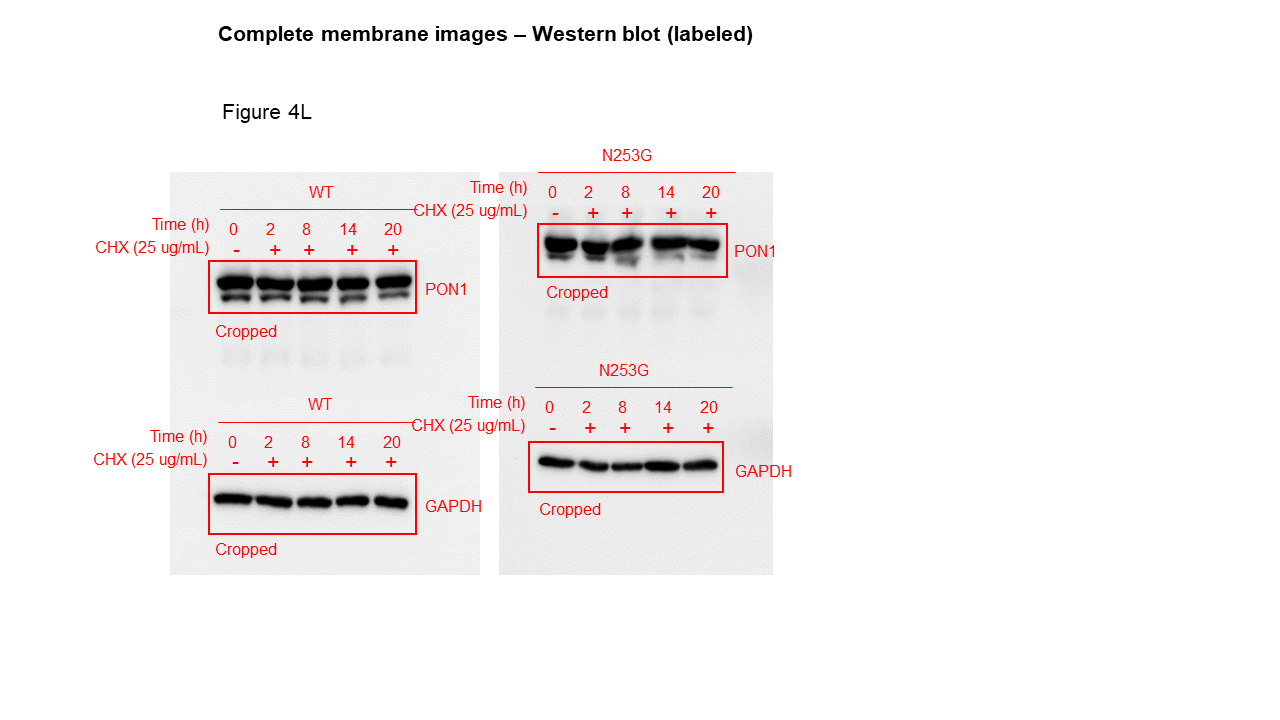

Supplement: Figure 4—source data 1. [file elife-75191-fig4-data1.zip › Figure 4/Figure 4L (labeled).tif]

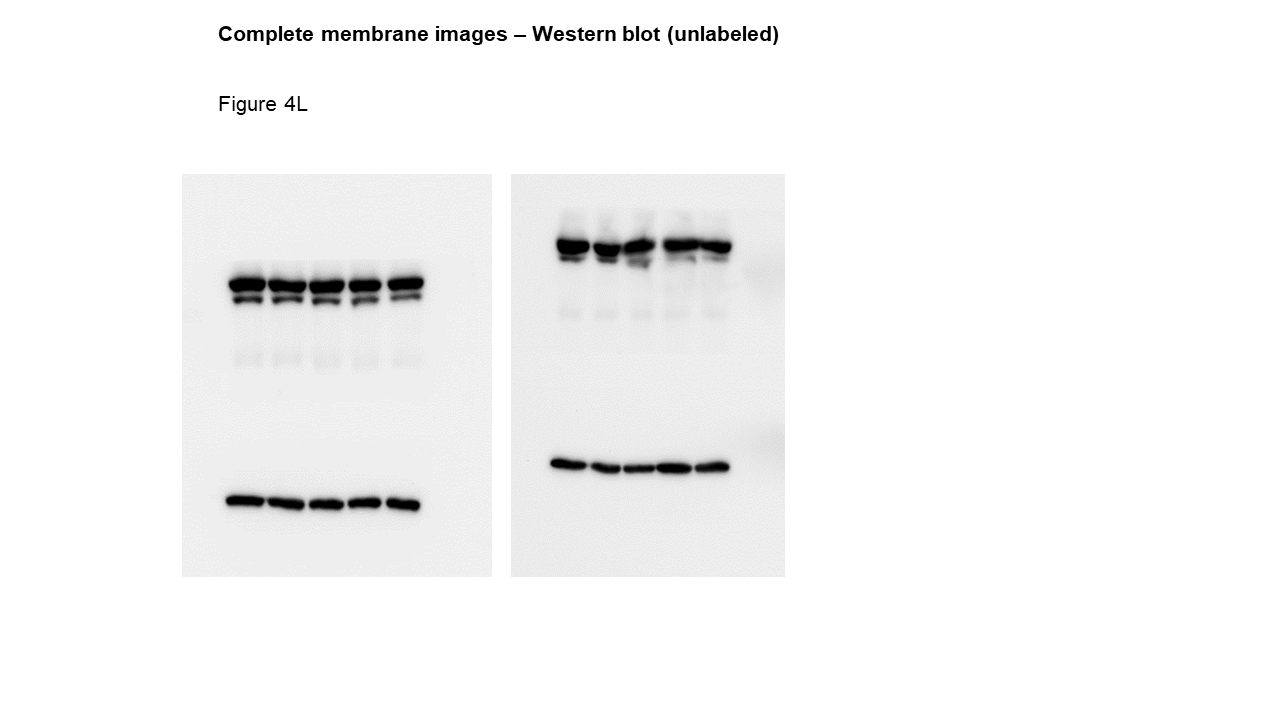

Supplement: Figure 4—source data 1. [file elife-75191-fig4-data1.zip › Figure 4/Figure 4L (unlabeled).tif]

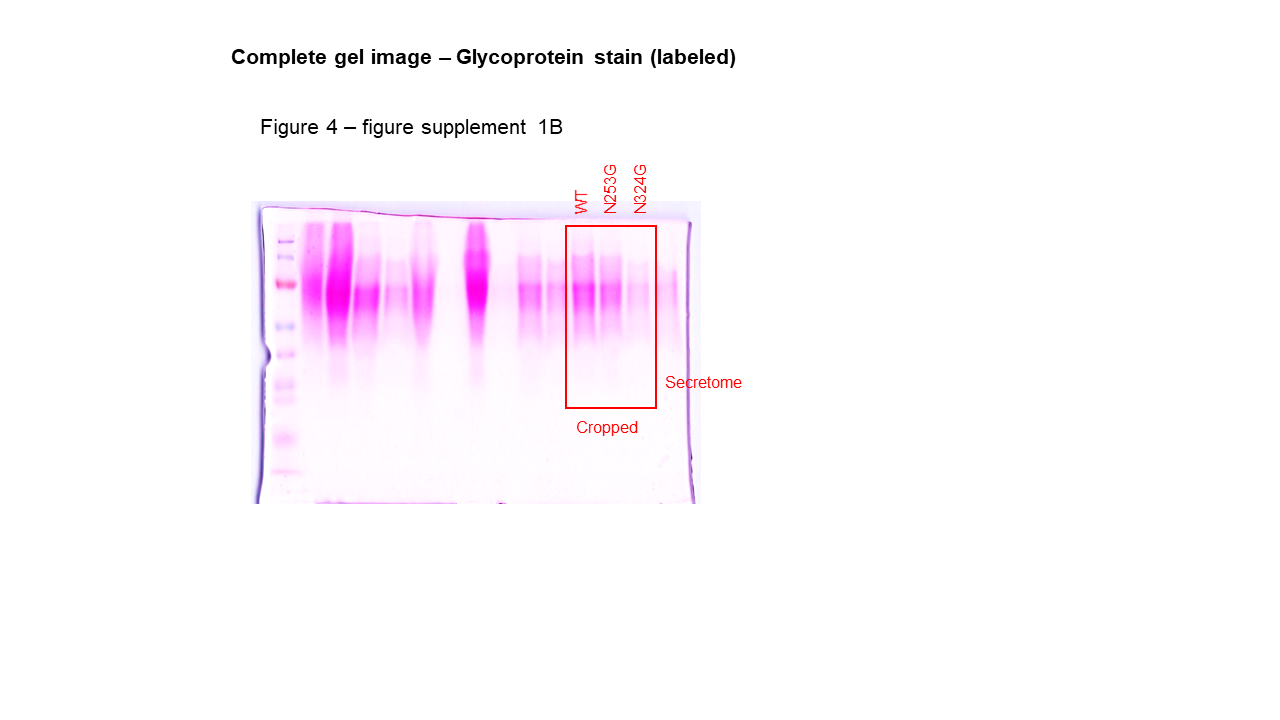

Supplement: Figure 4—source data 2. [file elife-75191-fig4-data2.zip › Figure 4-FS1/Figure 4_FS1B (labeled).tif]

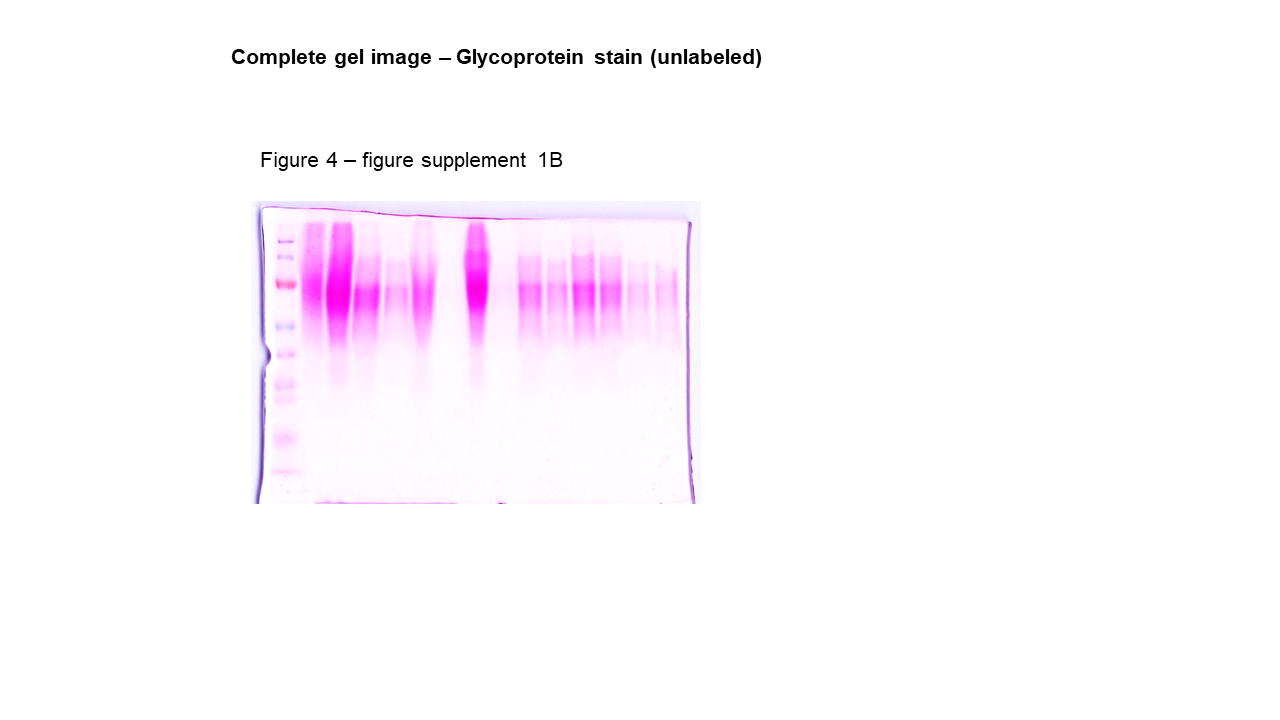

Supplement: Figure 4—source data 2. [file elife-75191-fig4-data2.zip › Figure 4-FS1/Figure 4_FS1B (unlabeled).tif]

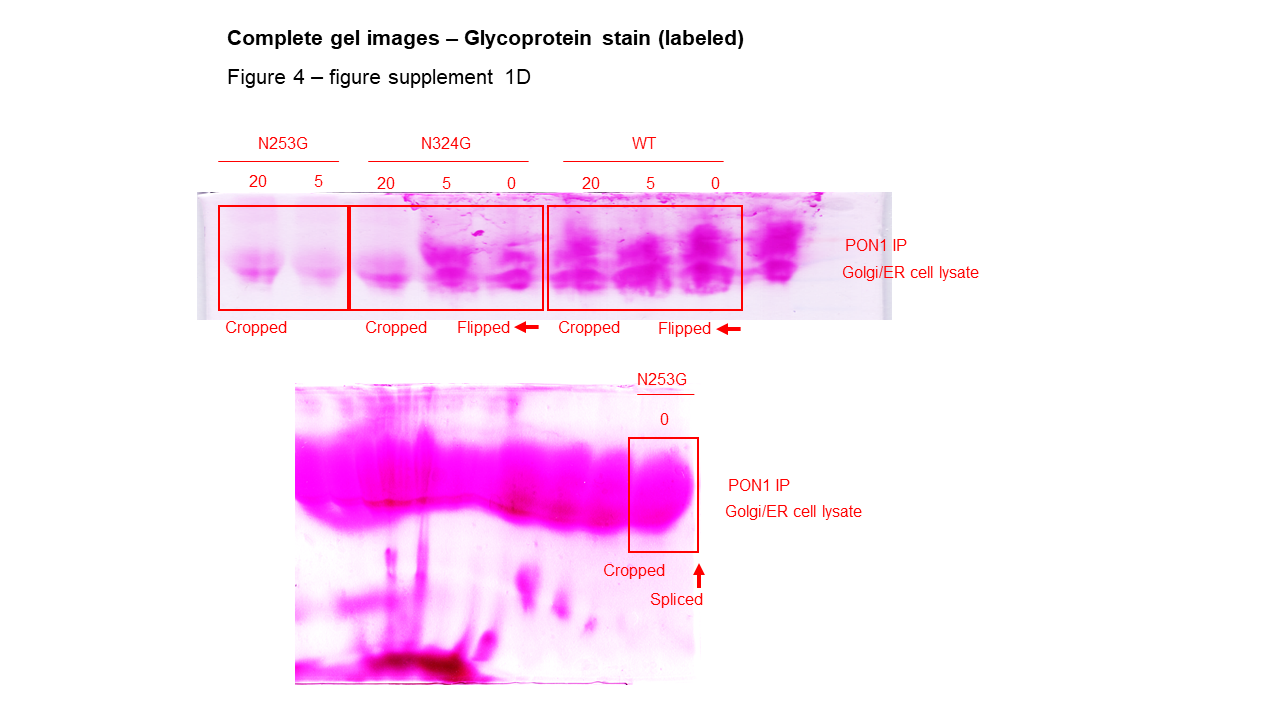

Supplement: Figure 4—source data 2. [file elife-75191-fig4-data2.zip › Figure 4-FS1/Figure 4_FS1D (labeled).tif]

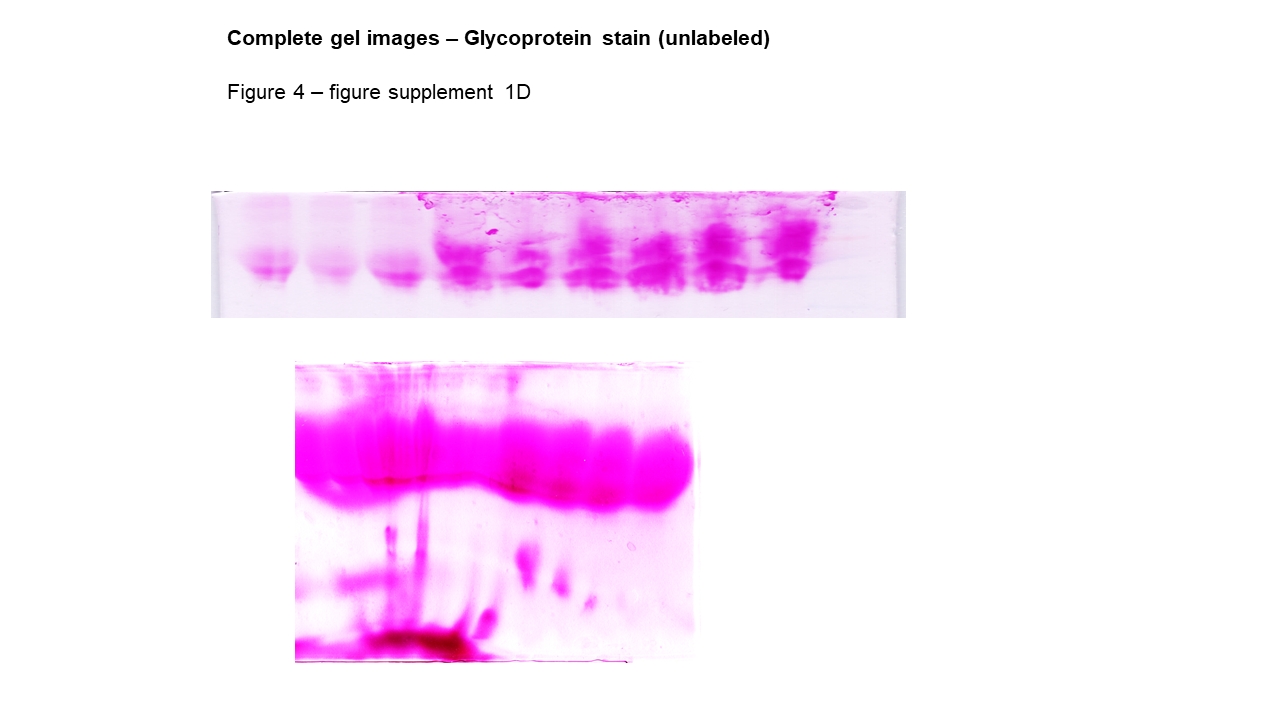

Supplement: Figure 4—source data 2. [file elife-75191-fig4-data2.zip › Figure 4-FS1/Figure 4_FS1D (unlabeled).tif]

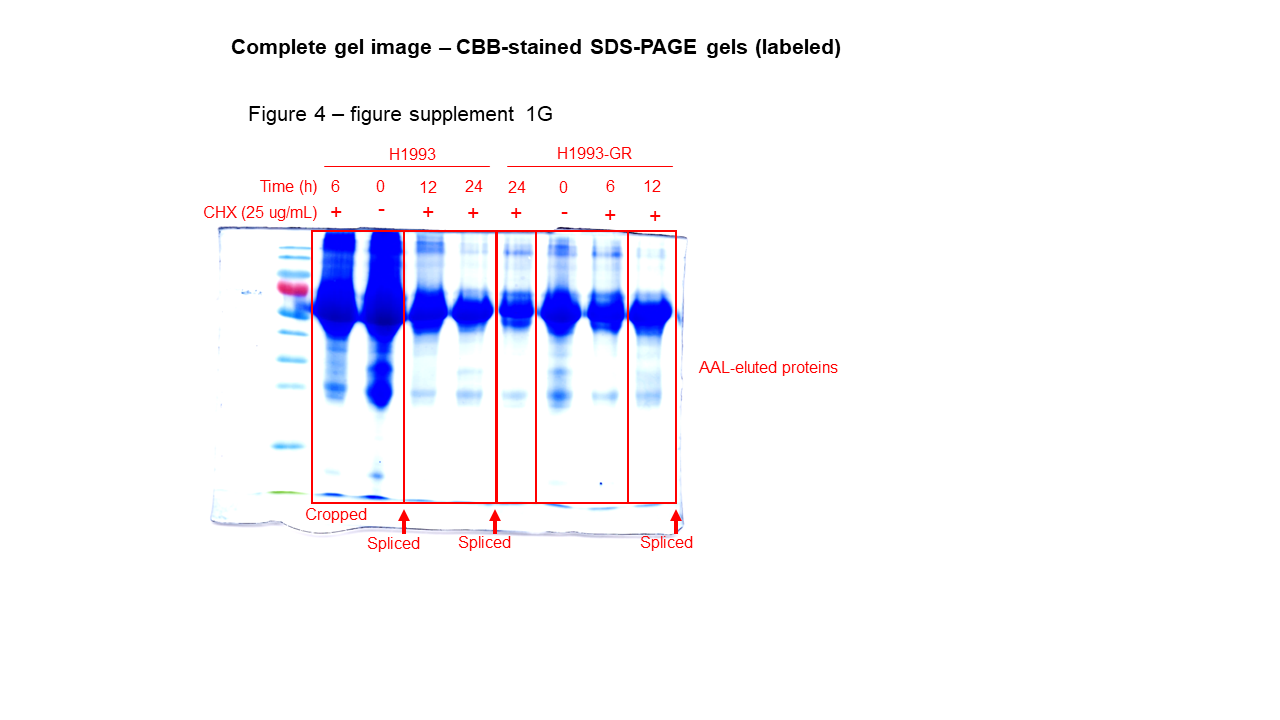

Supplement: Figure 4—source data 2. [file elife-75191-fig4-data2.zip › Figure 4-FS1/Figure 4_FS1G (labeled).tif]

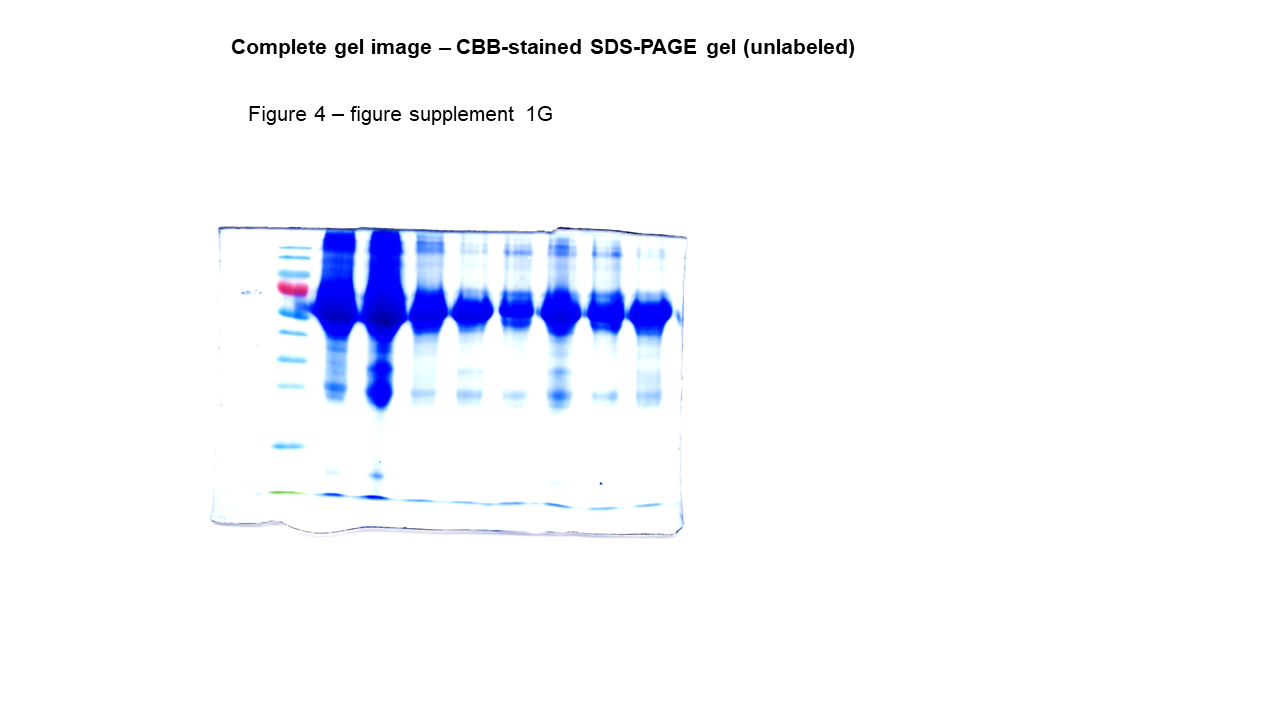

Supplement: Figure 4—source data 2. [file elife-75191-fig4-data2.zip › Figure 4-FS1/Figure 4_FS1G (unlabeled).tif]

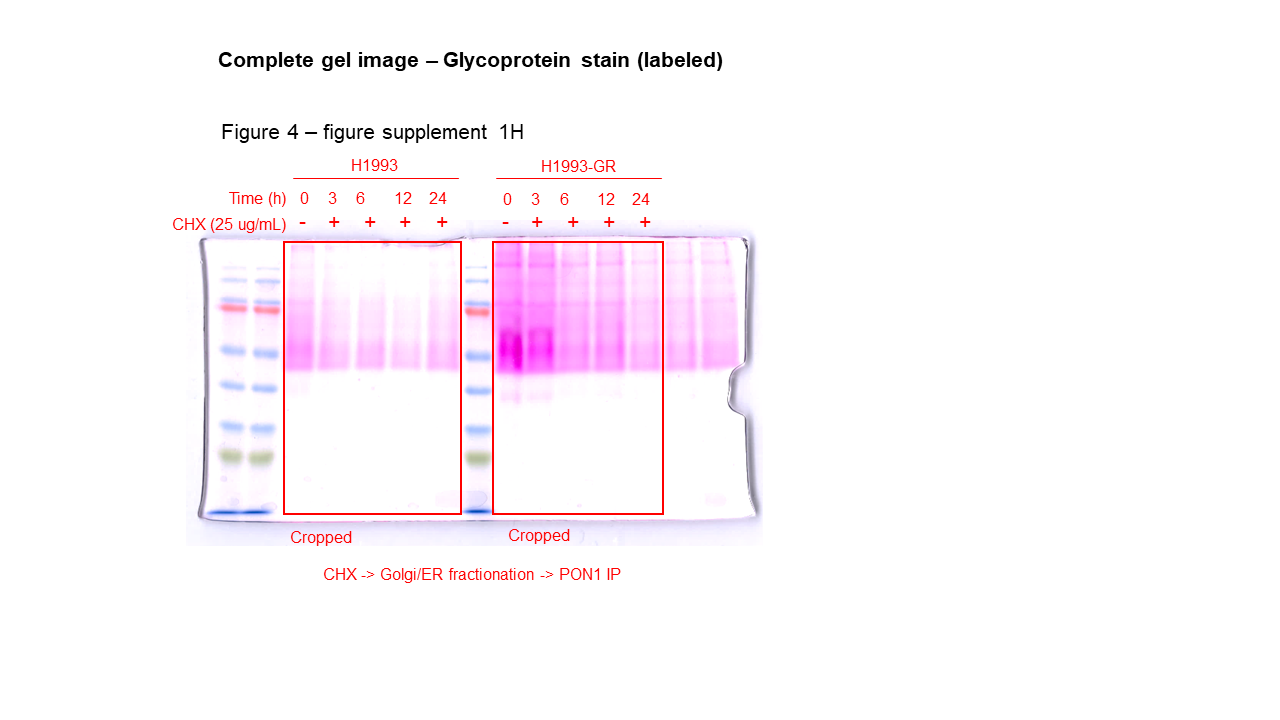

Supplement: Figure 4—source data 2. [file elife-75191-fig4-data2.zip › Figure 4-FS1/Figure 4_FS1H (labeled).tif]

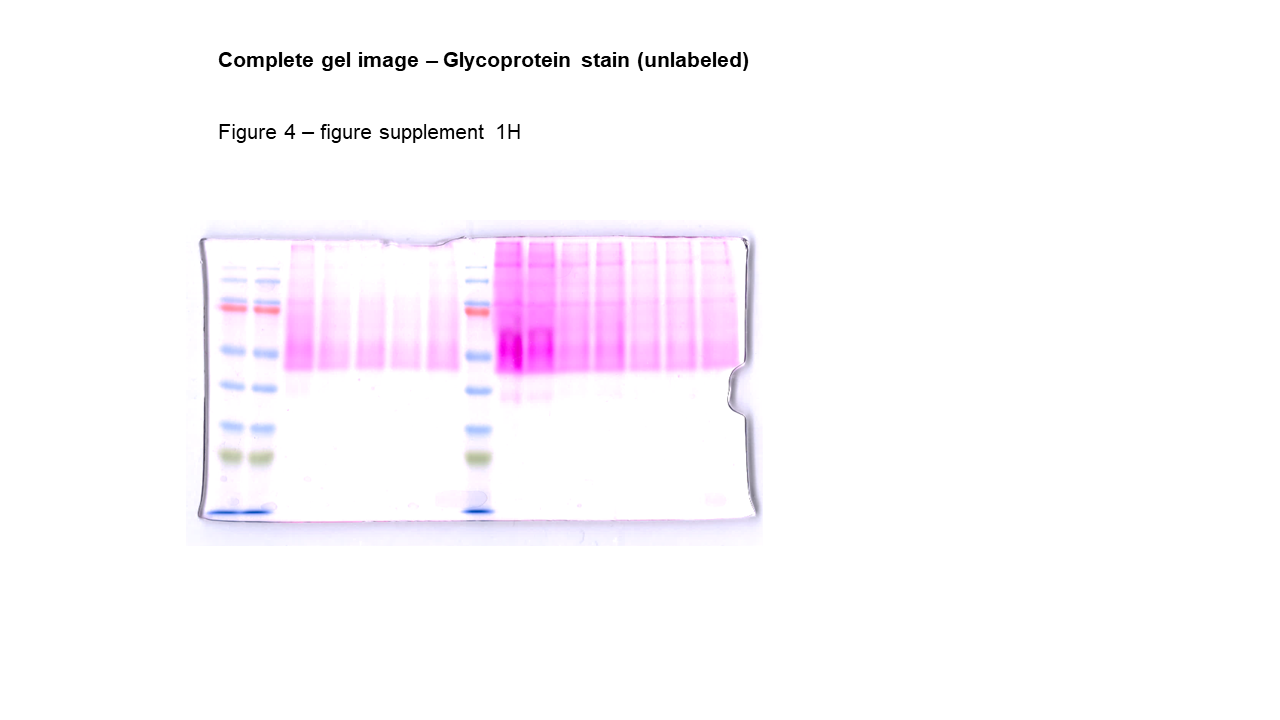

Supplement: Figure 4—source data 2. [file elife-75191-fig4-data2.zip › Figure 4-FS1/Figure 4_FS1H (unlabeled).tif]

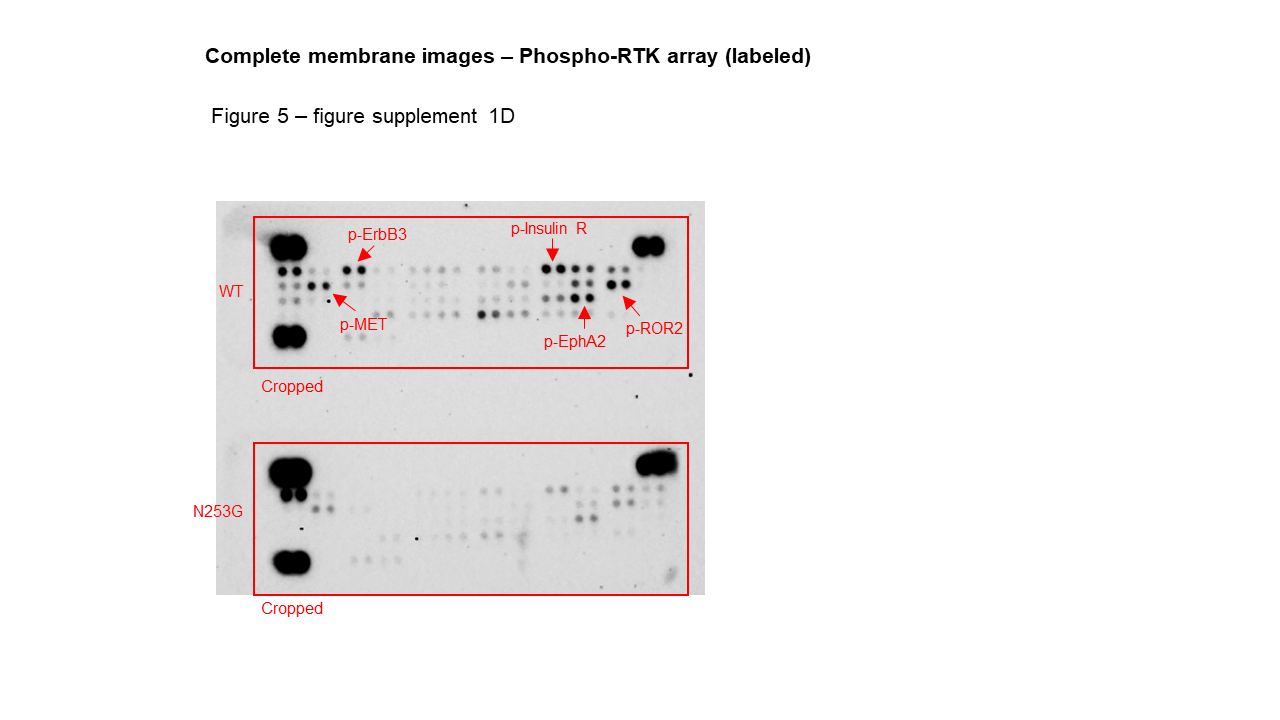

Supplement: Figure 5—source data 1. [file elife-75191-fig5-data1.zip › Figure 5-FS1/Figure 5_FS1D (labeled).tif]

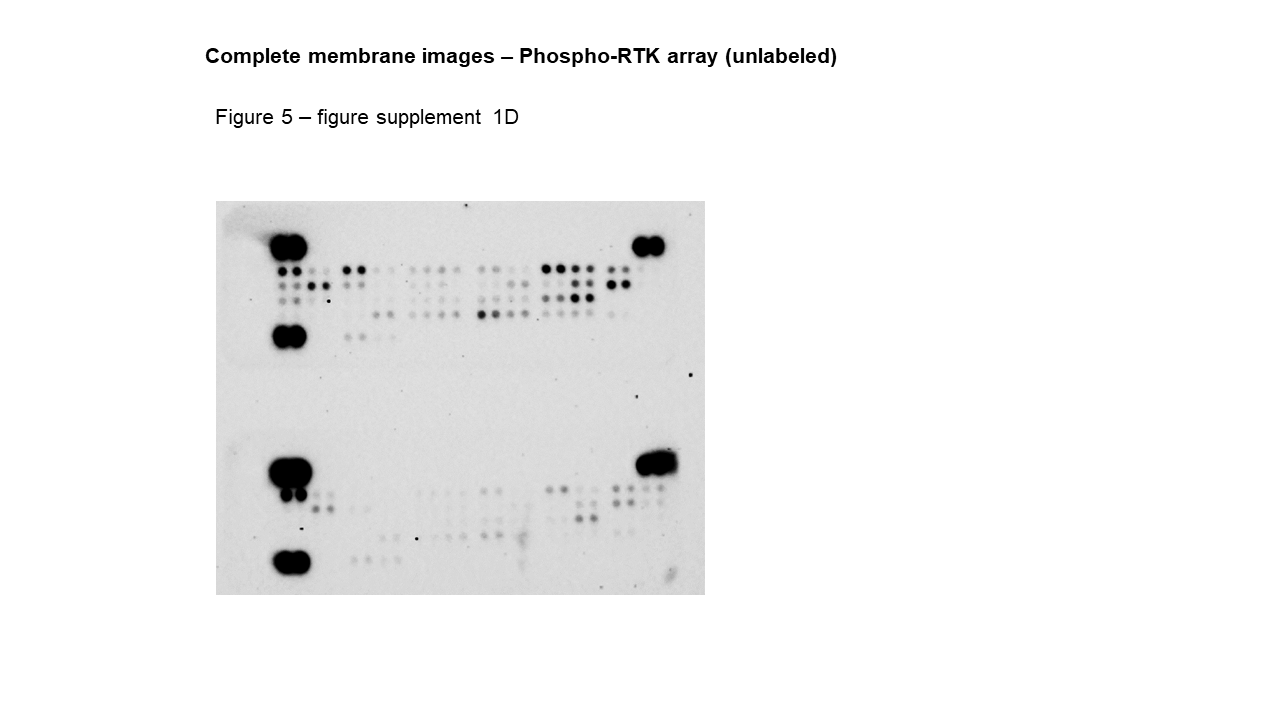

Supplement: Figure 5—source data 1. [file elife-75191-fig5-data1.zip › Figure 5-FS1/Figure 5_FS1D (unlabeled).tif]
